# Cross-study analyses of microbial abundance using generalized common factor methods

**DOI:** 10.1186/s12859-023-05509-4

**Published:** 2023-10-09

**Authors:** Molly G. Hayes, Morgan G. I. Langille, Hong Gu

**Affiliations:** 1 Department of Mathematics and Statistics, Dalhousie University, Halifax, NS Canada; 2 Department of Microbiology and Immunology, Dalhousie University, Halifax, NS Canada; 3 Department of Pharmacology, Dalhousie University, Halifax, NS Canada

**Keywords:** Cross-study analysis, Microbiome, Multi-group analysis, Common principal components, Common factor models, Ensemble principal component analysis

## Abstract

**Background:**

By creating networks of biochemical pathways, communities of micro-organisms are able to modulate the properties of their environment and even the metabolic processes within their hosts. Next-generation high-throughput sequencing has led to a new frontier in microbial ecology, promising the ability to leverage the microbiome to make crucial advancements in the environmental and biomedical sciences. However, this is challenging, as genomic data are high-dimensional, sparse, and noisy. Much of this noise reflects the exact conditions under which sequencing took place, and is so significant that it limits consensus-based validation of study results.

**Results:**

We propose an ensemble approach for cross-study exploratory analyses of microbial abundance data in which we first estimate the variance-covariance matrix of the underlying abundances from each dataset on the log scale assuming Poisson sampling, and subsequently model these covariances jointly so as to find a shared low-dimensional subspace of the feature space.

**Conclusions:**

By viewing the projection of the latent true abundances onto this common structure, the variation is pared down to that which is shared among all datasets, and is likely to reflect more generalizable biological signal than can be inferred from individual datasets. We investigate several ways of achieving this, demonstrate that they work well on simulated and real metagenomic data in terms of signal retention and interpretability, and recommend a particular implementation.

**Supplementary Information:**

The online version contains supplementary material available at 10.1186/s12859-023-05509-4.

## Background

Communities of microbes inhabit all areas of the environment, including the body cavities and exterior surfaces of larger organisms. A microbiome can be thought of as a community of shared genes and metabolic pathways that acts as a complex system in ways defined in part by their composition, or which microbial taxa are present and in what abundance [7]. These communities vary widely in composition, and certain patterns of colonization seem characteristic across individuals—and analogously across environmental sites—given similar conditions. In fact, mounting evidence suggests that micro-organisms comprising the gut microbiome interact with host systems in myriad ways, and that understanding these associations could have profound implications for our ability to predict, diagnose, or treat pathologies [6, 31, 39, 41]. Similarly, the environmental microbiota, such as the communities characterizing a particular stratum of the soil or a particular water column, have enormous influence on local physiochemical conditions with far-reaching implications for ecology, agriculture, fisheries, and biotechnology. Moreover, the study of micro-organisms, their community dynamics, and their interactions with the cellular systems of their hosts continues to help researchers elucidate the origin of life on Earth.

Both 16S rRNA gene sequencing (be it in the form of operational taxonomic units or amplicon sequence variants) and metagenomic sequencing are the two most common high-throughput next-generation sequencing technologies, followed by bioinformatic algorithms that allow researchers to ultimately obtain counts of the observed representatives of each microbial taxon in each sample to characterize the composition of the microbiome. Regardless of sequencing protocol, the output comprises sequences of base pairs from a random sample of the total collection of genes in the community. This means that we only observe a count of each taxon in a sample, and not its true abundance in the community (sampling errors). In addition, the “depth” of sequencing, or the average number of reads in a sample that align to a known reference, varies significantly by sample and is thus a source of multiplicative error on the counts. Since many short fragments of a sequence have to be read and aligned with each other in order for that sequence to be recognizable, samples with lower sequencing depth have lower observed counts and more uncertainty (sequencing depth issues). A common practice to deal with the sequencing depth issue is to use the relative abundance or proportions of taxa in a sample for statistical analysis, which causes the problem of spurious correlations when distributions of taxa are unbalanced (compositional effects) [2]. Furthermore, thousands of microbial taxa can be present in a single sample, many of which are present in extremely low numbers, while the number of samples is—as in any experiment—limited by practical constraints such as cost and participation. As a result, each dataset has far more features than samples [24], and in a given sample there will be zero instances of many taxa (high dimensionality and sparsity), and the marginal distributions of most of the taxa are very skewed (long tailed distributions). These difficulties invalidate most existing data exploration methods, such as principal component or factor analysis, for inferring factors associated with large variation among conditions [37]. Despite the promise held by microbial abundance data, analysis is so statistically challenging [43] that scope for application is currently limited.

For sequencing depth issues, microbial abundances have often been modelled as if they were continuous by computing proportions of observed counts to the read depth of the sample (we will call this “relative abundance” data). Some workflows instead rarefy counts, which sacrifices observed data in order to equalize read depth. Hence, two generally acknowledged (but not necessarily enacted) recommendations for better statistical treatment of abundance data are 1) that an appropriate discrete generating distribution be used to model the sampling of counts, such as Poisson, negative binomial, or multinomial, and 2) that sequencing depth error be treated within a statistical framework [29]. Additionally, it is well supported in all fields of ecology that the logarithmic scale can be useful when modeling populations of organisms in a community [33].

There are two available approaches—each of which assumes that the observations are conditionally Poisson-distributed—that can address all the above data issues and provide a good variance estimator. The first approach is the Poisson log-normal PCA (PLNPCA) [11], a fully parametric model in which the latent log Poisson means are assumed to follow a multivariate normal distribution, and the observed counts are—given the log means—independently Poisson-distributed. Sequencing depth can be treated as an offset in this model. The second approach is Poisson measurement error-corrected PCA (PoissonPCA) [22], which also assumes that the observed counts are—given the latent Poisson means—independently Poisson-distributed, but estimates an unbiased covariance matrix non-parametrically for any nonlinear transformation of the latent means, including the log-transformed case. The sequencing depth is dealt with by assuming a nuisance additive random variable on the log-scaled Poisson means. Both approaches model the counts by a mixture of Poisson distributions, where for PLNPCA the mixing distribution of the Poisson means is log-normal, and for PoissonPCA it is unspecified. Because the Poisson means vary between samples, the marginal distributions of counts are both over-dispersed and sparse to a similar extent to real data (detailed comparisons between the simulated data and the real data are given by Kenney et al. [22] in Section 7.3 and Supplementary Appendix F.4).

However, as it happens, further complications arise when genomic samples from different studies are compared with one another. In order to sequence DNA, it first has to be isolated from a sample, fragmented, and potentially amplified. Each of these processes requires a number of laboratory techniques and reagents, and procedures vary substantially between labs. Sequencing platforms also differ, and presumably there are also machine calibration differences between two sequencers of the same model. The result is that when two studies of similar design look at samples of similar origin, or even when identical samples are sent to two different labs for sequencing, the signal patterns are very different due to dominating “batch effects”. This noise persists even under highly controlled conditions [34] and can obscure the signal of interest: for example, machine learning classifiers enjoying good within-study performance may become grossly inaccurate when applied cross-study [38]. Batch effects impair our ability to determine whether results generalize to other cohorts, and preclude meaningful validation and meta-analysis [8, 26, 30].

In the RNA microarray literature, several approaches to correcting batch effects have been proposed [9]. The most popular of these, ComBat [21], performs gene-wise Bayesian location-scale adjustment. Several methods that combine regression and singular value decomposition have also been proposed, such as surrogate variable analysis [25] and RUV-4 [17], aiming to project away noise, which is identified as such based on gene expression signatures gleaned from regression. However, microarray data are very different from microbial abundance data; critically, we have no equivalent of “housekeeping genes” with which to base inferences about signal source. With the goal of pooling data across case–control microbial abundance studies, Gibbons et al. [18] proposed a within-study non-parametric normalization technique in which abundance of taxa in case samples are converted to percentiles of the abundance of equivalent taxa in control samples. However, their results are based on naive relative abundance models, which were run on a subset of taxa chosen in an ad hoc fashion (i.e., those that occurred in at least one third of case samples or one third of control samples). Multi-study factor analysis (MSFA) [12] and Bayesian MSFA [13] extend classical factor analysis to multiple groups to decompose features into factors that reflect shared vs. group-specific variability. However, like classical factor analysis, these methods decompose the naive sample variances of the observed data, which cannot capture the covariance structure of microbial abundances. Argelaguet et al. [5] proposed MOFA+, a multi-group multi-omics Bayesian factor analysis method that can consider multiple data types simultaneously in addition to multiple studies or sample groups with potential applications on assaying cells from multiple samples or conditions. But MOFA+ (like its predecessor, MOFA [4]) is unsuitable for microbial abundance data due to the use of a transformation in the Poisson case ($\Lambda (x)=\log (1+exp(x))$). This transformation is approximately linear when the Poisson mean is large, and thus the distribution is still long-tailed after the transformation. Most recently, Liu et al. [28] performed multi-group decomposition of correlations estimated by latent Gaussian copula models, but again this method lacks the machinery to address count data with multiplicative error.

This brings us to our purpose, which is to address the broadly meta-analytic difficulties presented by batch effects or technical variation in high-throughput microbial abundance data. We propose using a two-step ensemble method, with the first step obtaining estimated covariance matrices for each dataset by PoissonPCA or PLNPCA, and the second step to simultaneously decompose them to find a *q*-dimensional basis that is common to all groups, by common principal components analysis (FCPCA) [15], stepwise CPCA (SCPCA) [42], and MSFA [12]. This paper compares a number of ensemble methods on both simulated and real data and makes recommendations on the best candidate methods. In cases where the shared common signals across multiple data sets mainly represent biological variation, the proposed ensemble methods provide a better chance than existing methods of uncovering the underlying biological signals from the noisy data.

## Results and discussion

### Simulation study

We performed simulation studies of two synthetic groups of multivariate Poisson log-normal observations across several scenarios. These scenarios differed on the true signal (including the number—*q*—of eigenvectors that were common to both groups’ variance-covariance matrices, and whether the eigenvalues of the variance-covariance matrices were simultaneously decreasing), the sample sizes $n_1$ and $n_2$, whether or not the sample sizes were balanced, and whether or not sequencing depth correction was performed in the variance estimation stage. For each scenario, we report the average results over 100 replicate data sets. Our simulation methods could generate data very similar to the true observed microbiome data according to the sparsity, skewness and marginal distributions. See the Methods section for more details of the simulation design and simulation method.

**Fig. 1:**
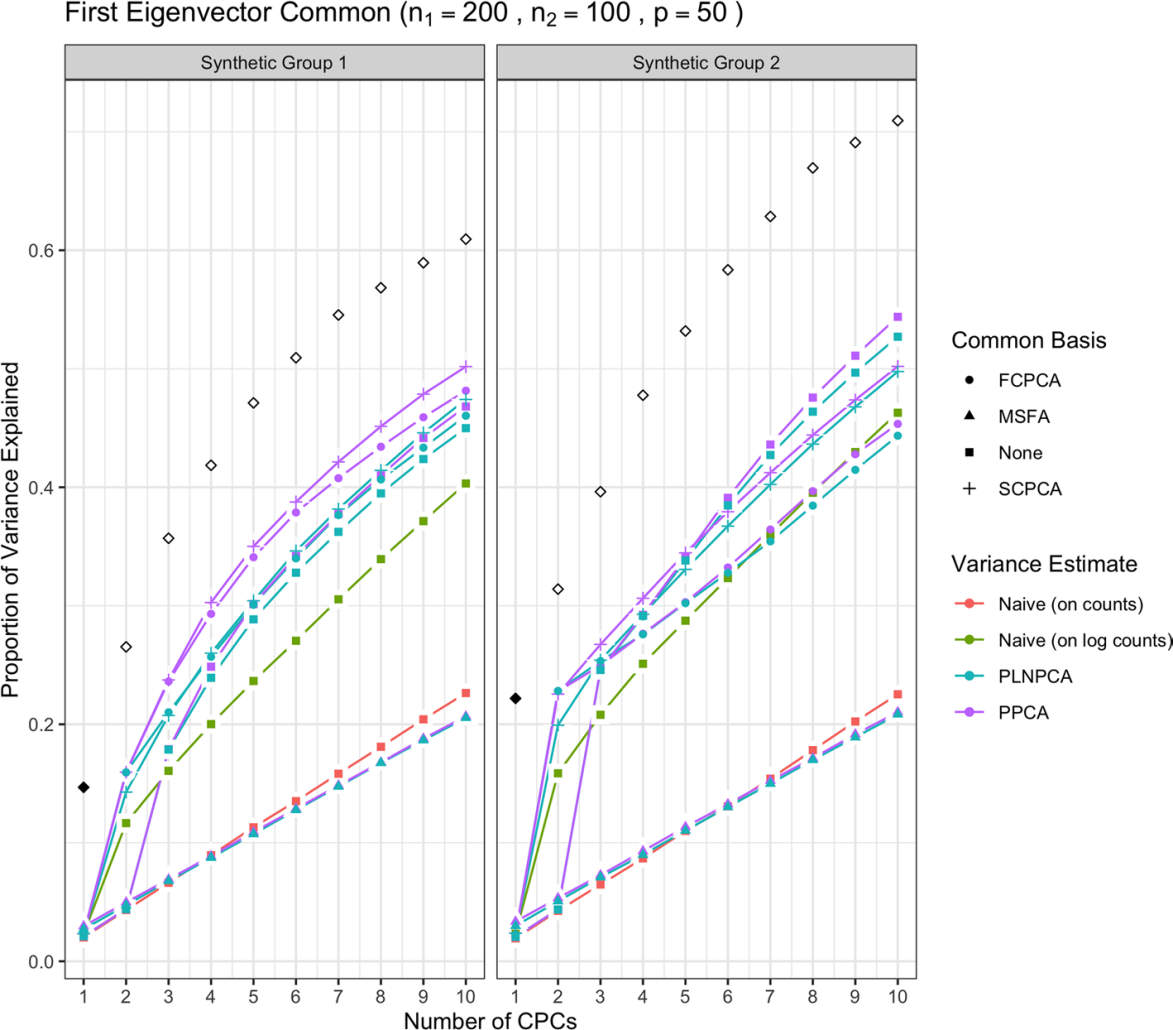
Simulation results for decreasing eigenvalues and one common eigenvector, with no sequencing depth correction; *p*=50, $n_1=200$, $n_2 = 100$. “None” as a common basis label means that Group 1 and Group 2 data were concatenated prior to variance estimation. The true common variances are in solid black; the true unique variances are in black outline-only

We now describe the results of the candidate ensemble methods listed in Table 1 and some single-group alternatives, which we ran on the simulated data. The single-group methods were all run on the data from the two groups concatenated together, and these methods comprised PoissonPCA, PLNPCA, naive PCA on counts, naive PCA on log counts, naive PCA on relative abundances, and naive PCA on log relative abundances. Representative results of the simulations are given by Fig. 1 through Fig. 5, where in each plot the true eigenvalues are given in black. We show here results for small, unbalanced sample sizes ($n_1=200$, $n_2=100$) only, which have the most relevance for real studies, while some results for larger sample sizes and balanced sample sizes are provided in the Additional file 1.

**Table 1:** The twelve candidate ensemble methods

	Ensemble methods	
	PoissonPCA + SCPCA	PLNPCA + SCPCA
No SDC	PoissonPCA + FCPCA	PLNPCA + FCPCA
	PoissonPCA + MSFA	PLNPCA + MSFA
	PoissonPCA + SCPCA	PLNPCA + SCPCA
SDC	PoissonPCA + FCPCA	PLNPCA + FCPCA
	PoissonPCA + MSFA	PLNPCA + MSFA

**Fig. 2:**
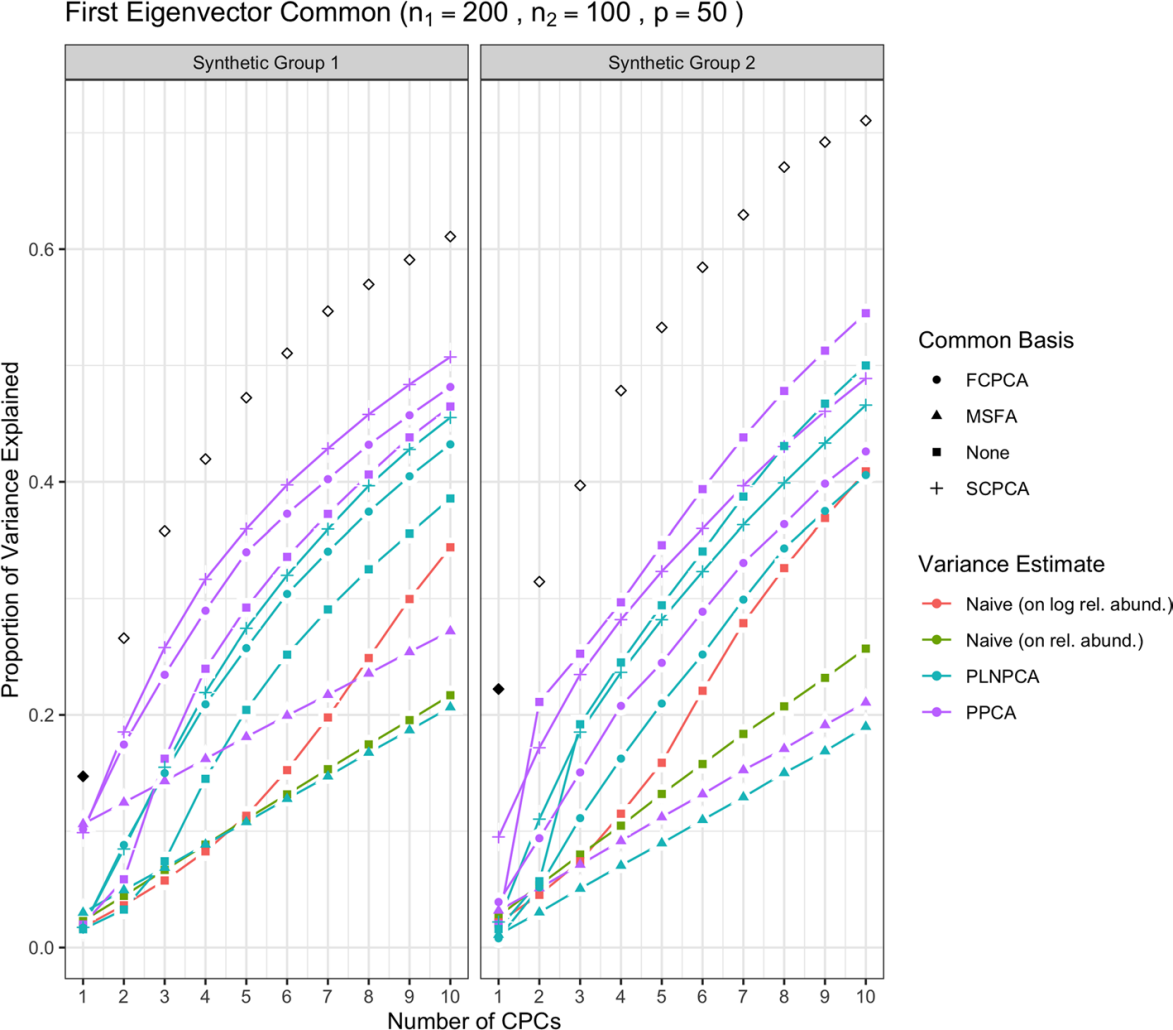
Simulation results for decreasing eigenvalues and one common eigenvector, with sequencing depth correction; *p*=50, $n_1=200$, $n_2 = 100$. “None” as a common basis label means that Group 1 and Group 2 data were concatenated prior to variance estimation. The true common variances are in solid black; the true unique variances are in black outline-only

**Fig. 3:**
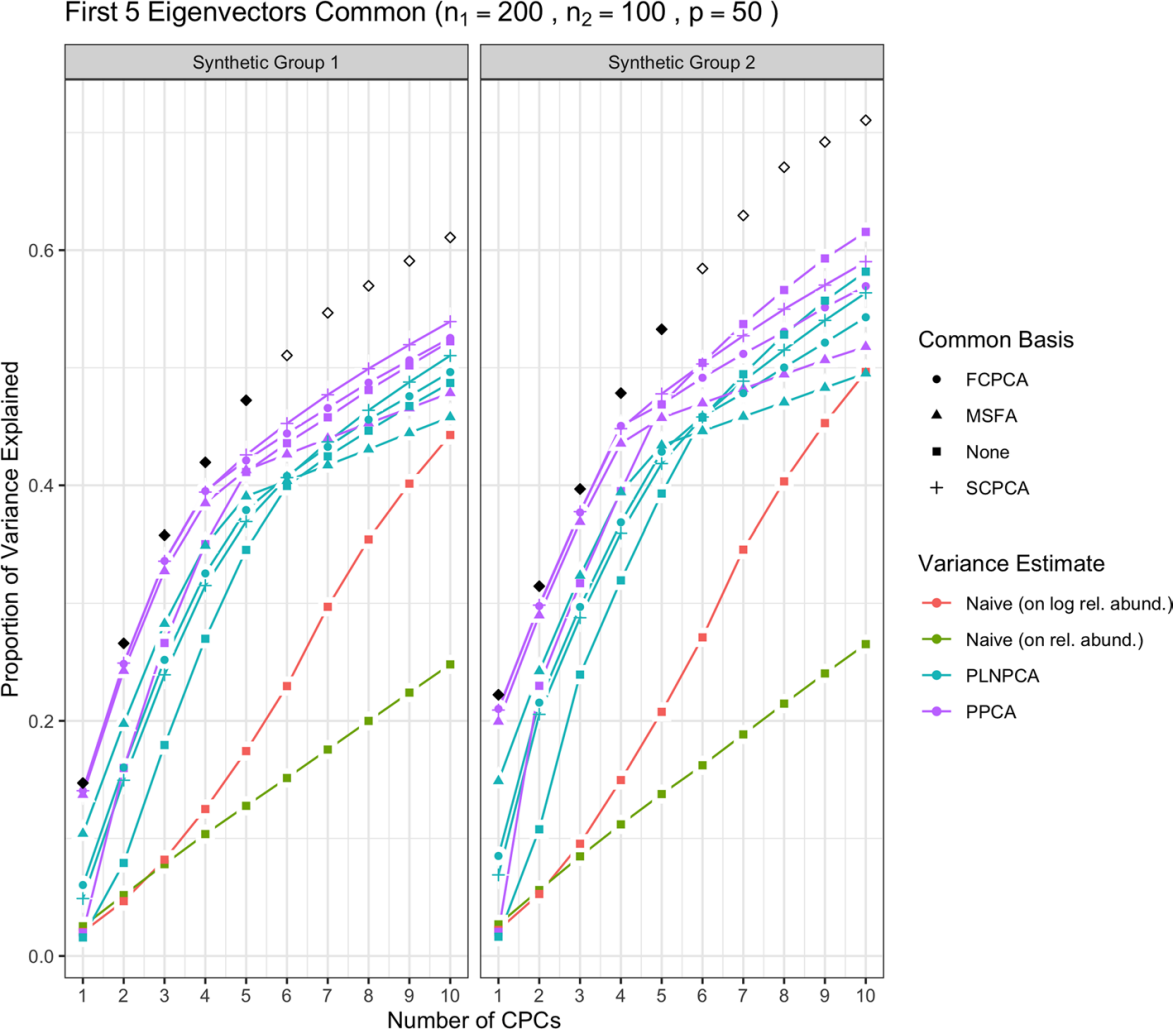
Simulation results for decreasing eigenvalues and five common eigenvectors, with sequencing depth correction; *p*=50, $n_1=200$, $n_2 = 100$. “None” as a common basis label means that Group 1 and Group 2 data were concatenated prior to variance estimation. The true common variances are in solid black; the true unique variances are in black outline-only

We first show the results for all methods under simulation conditions in which the variance-covariance matrices of Group 1 and Group 2 share only the first eigenvector and their eigenvalues are decreasing. In Fig. 1, the methods were run with no sequencing depth corrections applied: since all results without sequencing depth correction show similar patterns, we report this case only. The abbreviation “SDC” in tables and figures henceforth refers to “sequencing depth correction”. In Fig. 2, all methods were run under the same simulation conditions as the aforementioned but with sequencing depth corrections applied: that is, PoissonPCA with its compositional correction, PLNPCA with observed read counts as offsets, and the synthetic counts were transformed to synthetic relative abundances before application of the naive PCA methods. Figure 3 shows the results of the analogous scenario except that $\Sigma _1$ and $\Sigma _2$ shared the first five principal eigenvectors instead of only the first.

**Fig. 4:**
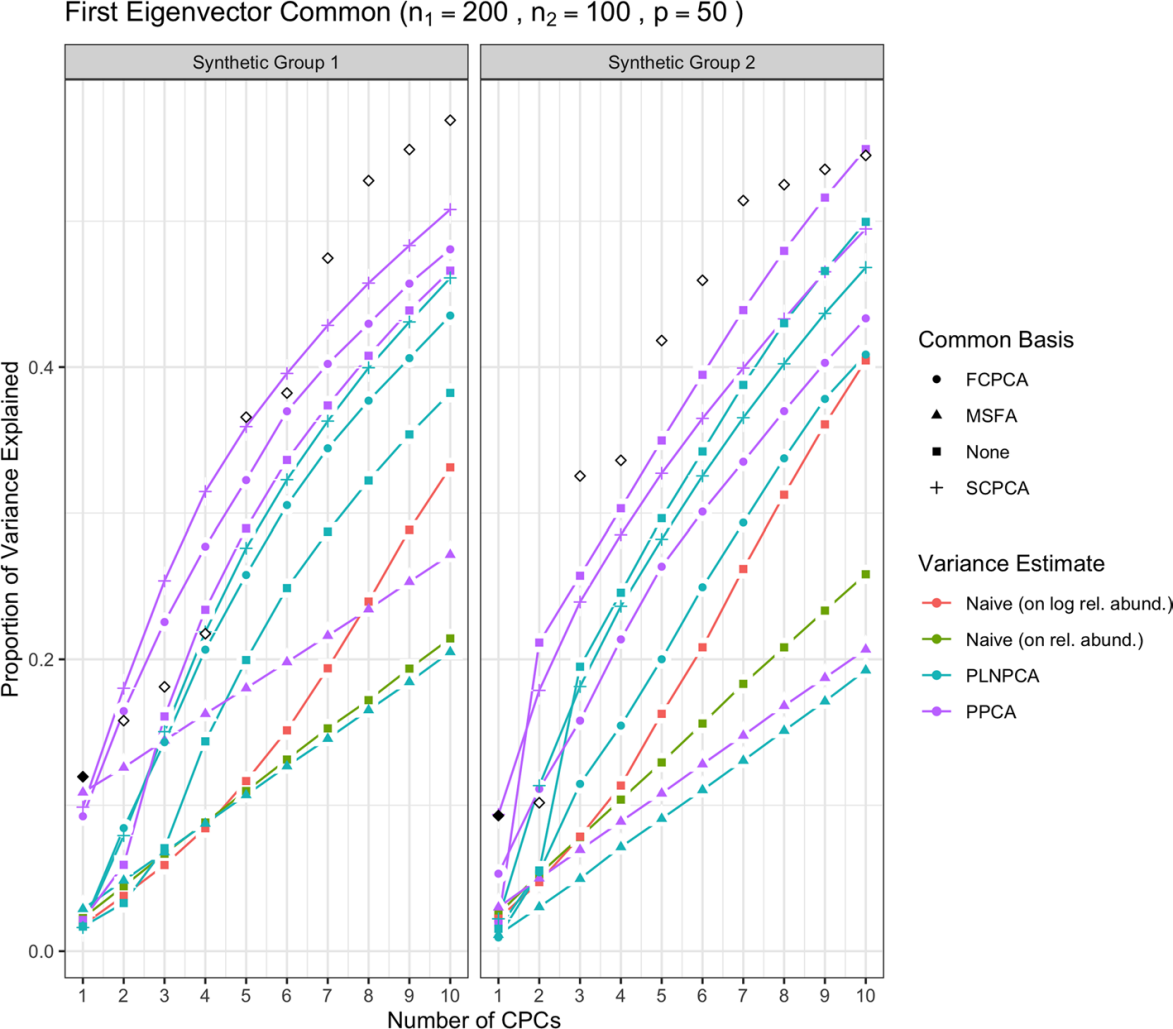
Simulation results for non-decreasing eigenvalues and one common eigenvector, with sequencing depth correction; *p*=50, $n_1=200$, $n_2 = 100$. “None” as a common basis label means that Group 1 and Group 2 data were concatenated prior to variance estimation. The true common variances are in solid black; the true unique variances are in black outline-only

**Fig. 5:**
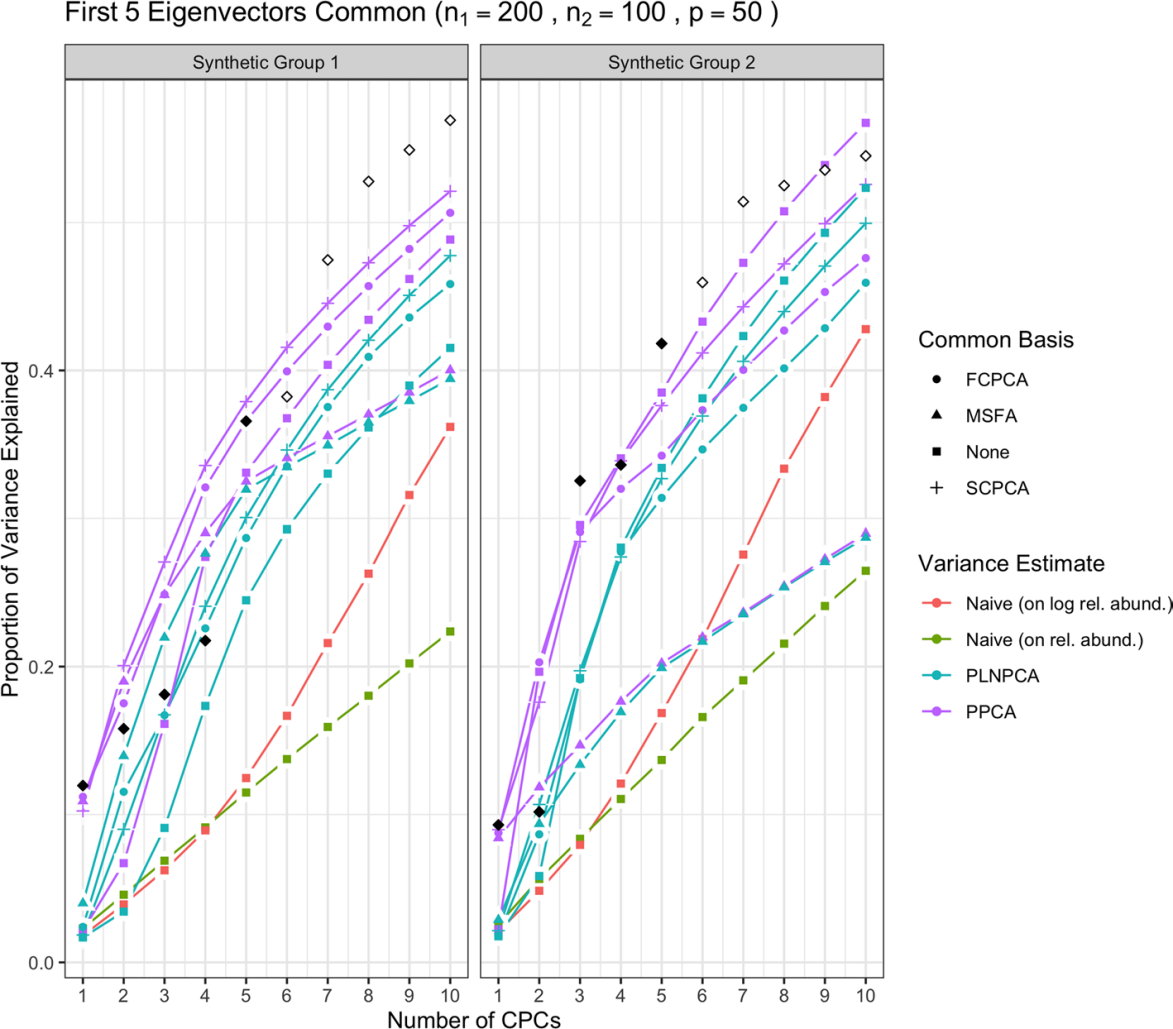
Simulation results for non-decreasing eigenvalues and five common eigenvectors, with sequencing depth correction; *p*=50, $n_1=200$, $n_2 = 100$. “None” as a common basis label means that Group 1 and Group 2 data were concatenated prior to variance estimation. The true common variances are in solid black; the true unique variances are in black outline-only

Then, Fig. 4 shows the results of the methods applied with sequencing depth correction to synthetic data for which the variance-covariance matrices of Group 1 and Group 2 shared one eigenvector, but with non-decreasing eigenvalues (i.e., the eigenvalues on the shared eigenvector are not the largest eigenvalues). Figure 5 shows the results for the analogous case except with $\Sigma _1$ and $\Sigma _2$ sharing five common eigenvectors instead of one. In each of these figures, the true variances (the eigenvalues used to simulate the data) associated with the *q* shared eigenvectors were plotted with solid black points, and subsequently the true variances associated with their unique eigenvectors were plotted with outline-only points.

From Fig. 1, we see that without sequencing depth corrections, all methods failed to capture the shared true variance on the first common principal component (CPC), and instead that axis captured the unique variation from sequencing depth. The second CPC then usually explained a large proportion of common variance. This was a typical pattern for all simulation scenarios when no sequencing depth correction was applied, which showed it is important to adopt methods with the sequencing depth correction in real data analysis, especially if there is obvious large variation in sequencing depth across samples.

Through Figs. 1, 2, 3, 4 and 5, we see both the naive PCA methods on relative abundances or raw counts for concatenated data were not able to find good rotations for the data. As a result, these methods showed very slow increases in their cumulative explained variance, with no improvement even as the number of shared eigenvectors increased. It is also obvious that the results of these two methods were much worse than the naive PCA methods on their corresponding log-transformed data. This is not surprising, because the true shared CPC directions should be calculated on the log scale instead of the original data scale. This misspecification of the model had a large impact on the resulting principal component directions. This also showed that the methods based on the covariance matrix estimated directly on the original microbiome data can fail badly in capturing the signals in the data, because microbiome data are very far from being normally distributed. See Additional file 1: Figs. S22 to S25 for comparisons of the PCA analyses based on covariance matrices obtained on the original scale versus the logarithmically transformed scale on real metagenomic data.

Compared to the naive PCA method on log relative abundances, Poisson PCA and PLNPCA applied alone on the concatenated data (labeled as “None” in all figures) showed much better results. This difference reflects the different treatments to the sequencing depth correction, zero counts, and Poisson measurement errors. The naive PCA method on log relative abundances is a method popularly used in microbiome data analysis. It usually first changes the zero counts to a small ad hoc number before taking the logarithm, which can generate bias. It then ignores the Poisson measurement errors, which typically leads to the estimated principal directions being far from the truth (see detailed comparisons of these three methods in Kenney et al. [22]). The simulations showed generally better performance by some ensemble methods based on PoissonPCA or PLNPCA, and by PoissonPCA or PLNPCA alone. This suggests that methods tailored to deal with Poisson measurement error should be the first choice among the different options.

PoissonPCA or PLNPCA alone on the concatenated data generally tended to capture a very small amount of common signal on the first CPC, but showed good recovery on the second or the third CPC. This is because the log Poisson means for the two groups of data each follow a multivariate normal distribution but with different mean vectors for the two groups. A common mean vector was estimated and subtracted in estimating the covariance matrix for the concatenated data. The first PC direction largely captured this mean difference, and thus when projecting the true variance of the data on this direction, it showed very small values.

Between PoissonPCA and PLNPCA, PoissonPCA is seen here to have consistently outperformed PLNPCA in terms of the reconstruction of the dominant signal in each group, despite the fact that the data were simulated under PLNPCA’s generative model. One possible explanation for this is that PoissonPCA’s moment-based variance estimator may be less sensitive than PLNPCA’s variance estimator to the outliers that arise from the Poisson log-normal generating process. Interestingly, the difference in performance between PoissonPCA and PLNPCA is negligible without sequencing depth correction, which suggests that PoissonPCA’s compositional correction to the variance estimate may be superior to PLNPCA’s use of observed read count as a model offset. Incidentally, PoissonPCA was also an order of magnitude faster to run in our simulations.

The ensemble methods dramatically outperform PoissonPCA or PLNPCA alone and the naive PCA methods on the concatenated data, especially on the estimation of the first eigenvector, and when some of the large eigenvalues are not associated with the shared eigenvectors. When the variance estimates from PoissonPCA or PLNPCA are decomposed using SCPCA, FCPCA, or MSFA, we observe highly desirable dimension reduction behavior. This provides evidence that our ensemble method holds value for extracting signal and biological insight from collections of noisy metagenomic and 16S datasets, which may well possess extensive unique variation.

For the estimation of the low-dimensional basis, the two CPCA methods are very similar and both did well. SCPCA appeared to outperform FCPCA when the signal was more difficult to resolve, such as when there were very few shared eigenvectors and the true cumulative variance increased slowly (e.g., Group 2 with one shared eigenvector). While technically SCPCA, FCPCA, and MSFA are all designed for positive-definite symmetric matrices, in practice SCPCA performed well even when the covariance matrix was indefinite, which occasionally occurred in the PoissonPCA case (see Methods section for details on how the PoissonPCA variance estimates were reconstructed to be positive-definite before running FCPCA and MSFA). MSFA, which assumes that only *q* axes are common among the *S* groups (unlike CPCA, which assumes that all *p* axes are common), performed well in some cases, with the caveat that we were only able to specify the true *q* because this was a simulation. However, when common signal was very low (*q*=1), MSFA struggled to capture any variation on the first axis for Group 2 compared to SCPCA. Moreover, the MSFA optimization routine sometimes failed, and the simulation results for MSFA had to be averaged over the successful replicates (typically around 90%, depending on the simulation scenario). In addition, MSFA had by far the slowest run-time—on the order of minutes, as compared to seconds for all others—and so from these simulations it would appear that the CPCA approaches have more practical utility, especially SCPCA.

Figures S1 through S8 show more scenarios, including balanced design with small sample sizes, and both balanced and unbalanced designs with large sample sizes. Across all scenarios, the figures clearly show that the PoissonPCA combined with SCPCA outperformed all other methods and would be the suggested choice. This result is especially clearly confirmed by the large sample simulations shown in Additional file 1: Figs. S1, S2, and S5 to S8.

**Fig. 6:**
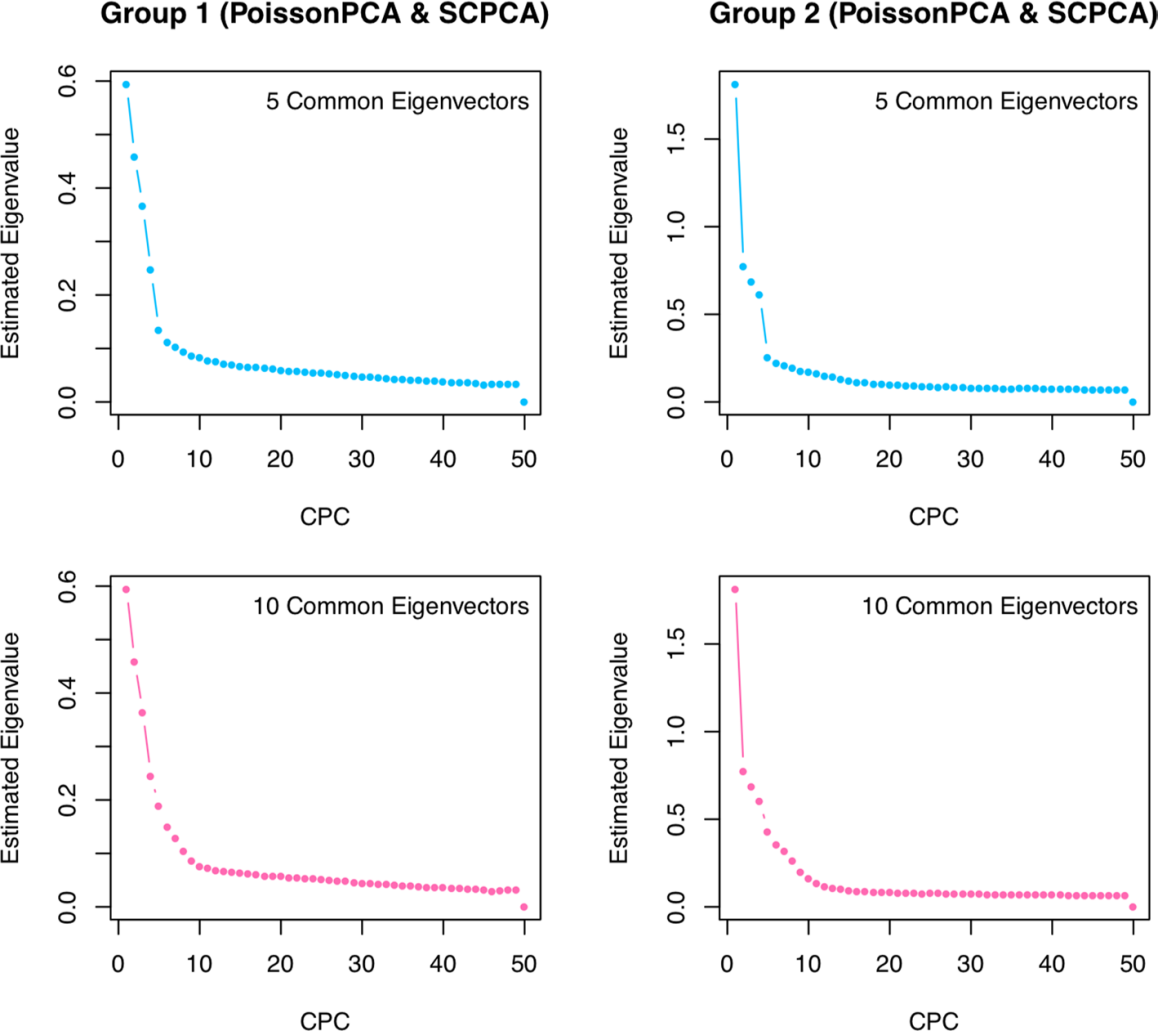
Scree plots of estimated eigenvalues from PoissonPCA and SCPCA for each group with 5 or 10 shared eigenvectors

finally, Fig. 6 contains scree plots for the estimated eigenvalues from PoissonPCA followed by SCPCA for each group, where $\Sigma _1$ and $\Sigma _2$ share either 5 or 10 common eigenvectors and the true eigenvalues are simultaneously decreasing. These plots show that the differences between successive estimated eigenvalues drop to near zero when the number of CPCs is larger than the true number of shared eigenvectors, which provides evidence that we will be able to make a good choice of *q* when applying the method to real data for which we cannot know the true number of shared eigenvectors.

### Colorectal cancer data analysis

It is not surprising that many studies have found links between the gut microbiota and cancer of the colon. To investigate the performance of our candidate ensemble methods on real data, we re-analyzed metagenomic datasets from Feng et al. [14] and Zeller et al. [47], each consisting of fecal samples from participants diagnosed with colorectal carcinoma (CRC) or non-malignant colorectal adenoma and from controls (study and participant characteristics are summarized in Table 2).

**Table 2:** Comparison of Zeller et al. [47] and Feng et al. [14]

	Zeller et al. [47]	Feng et al. [14]
Number of samples	199	154
Country of origin	France, Germany	Austria
Sequencing technology	Illumina HiSeq	Illumina HiSeq
Number of CRC samples	91	46
Number of adenoma samples	42	47

**Fig. 7:**
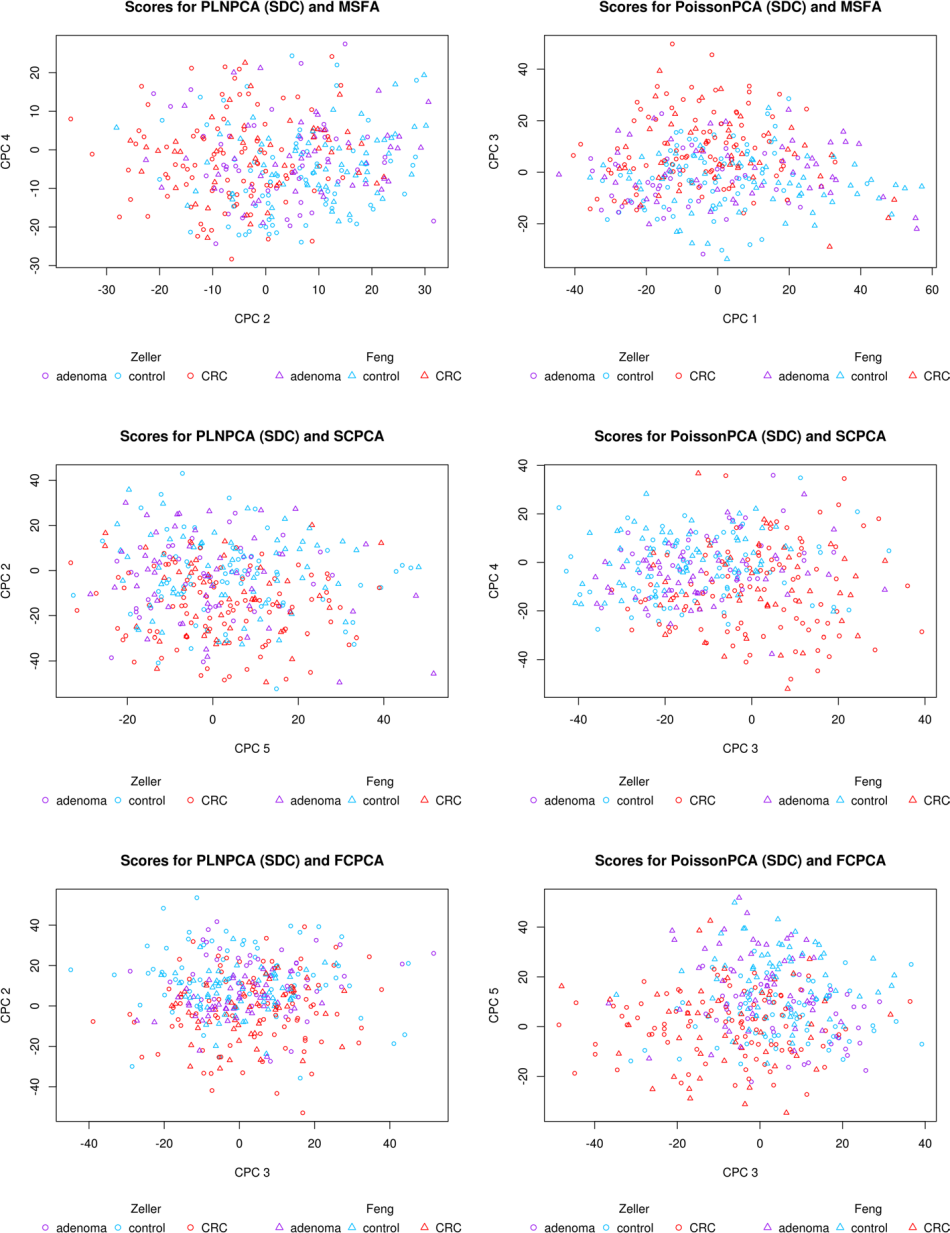
Scores from ensemble methods with SDC by disease state

**Fig. 8:**
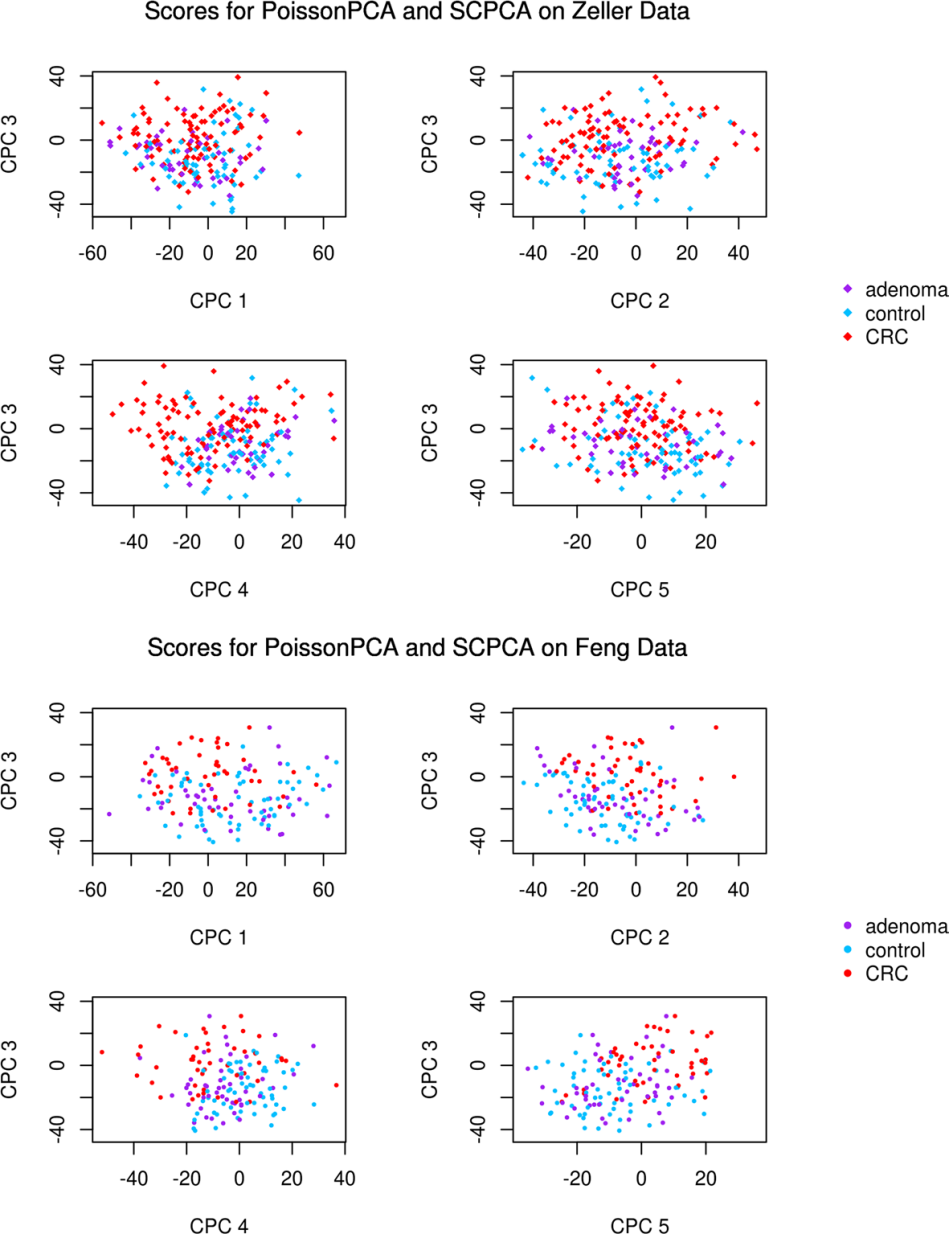
Score plots by disease state (PoissonPCA with SDC and SCPCA)

**Fig. 9:**
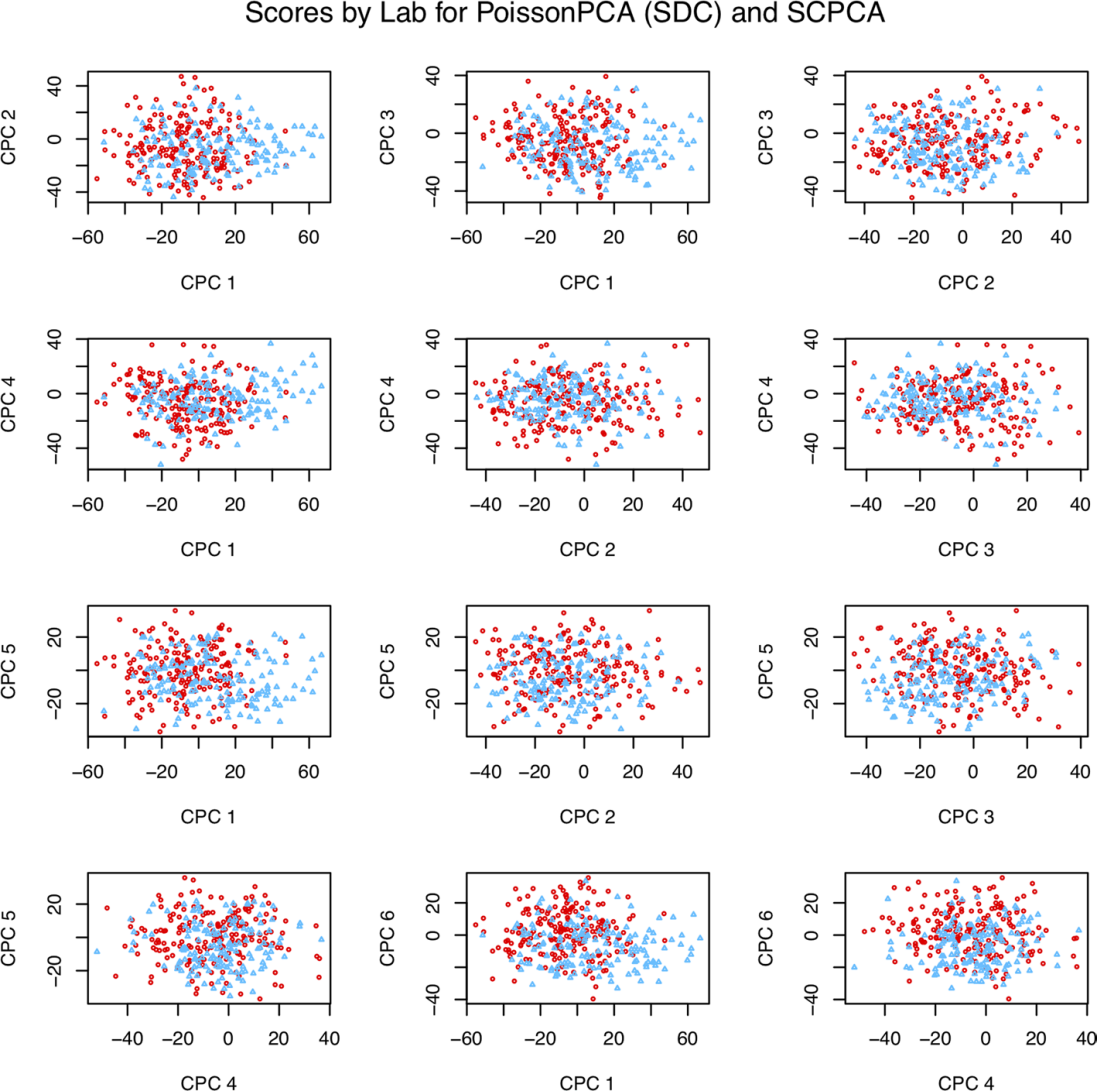
Scores by study of origin (PoissonPCA with SDC and SCPCA)

**Fig. 10:**
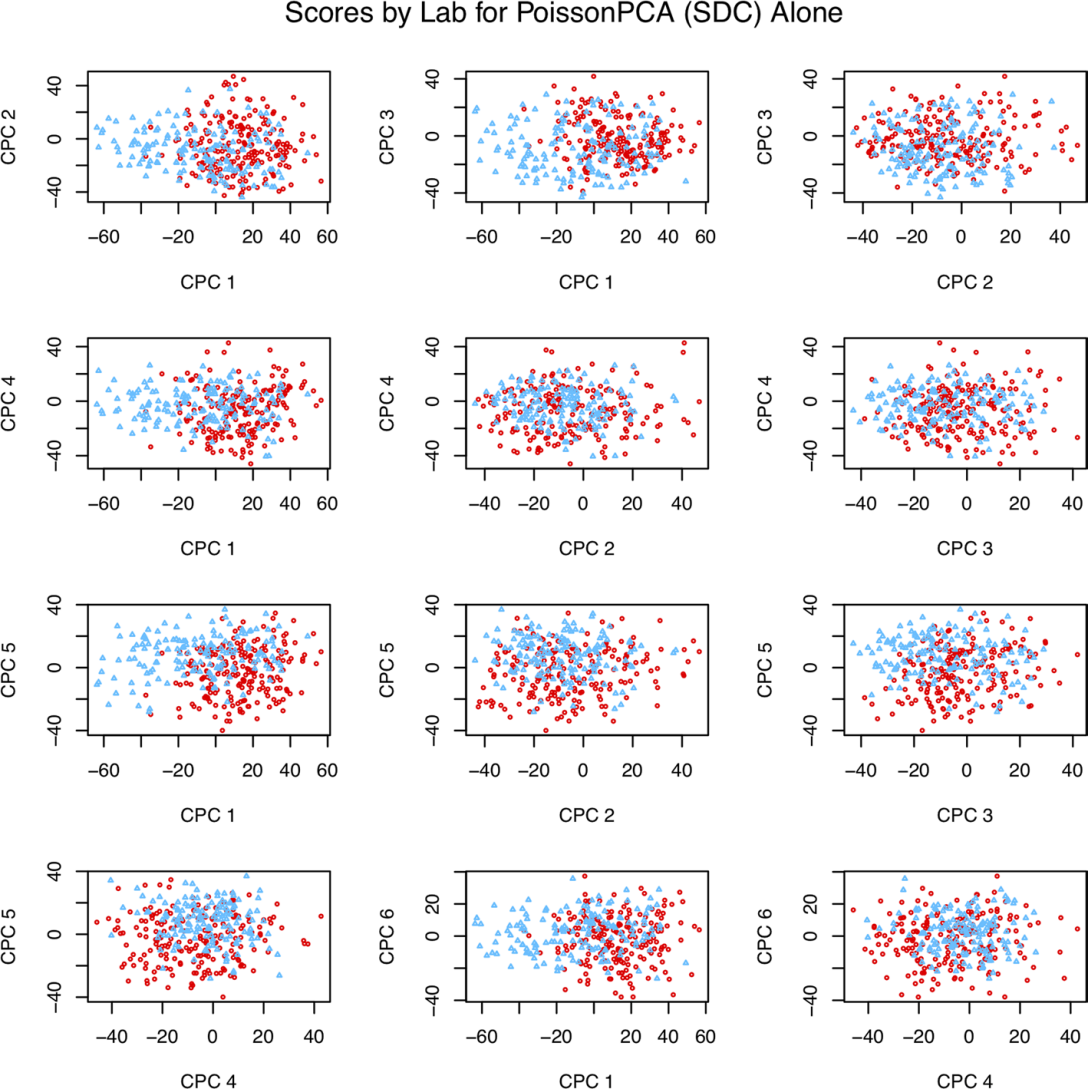
Scores from PoissonPCA alone with SDC by study of origin

**Fig. 11:**
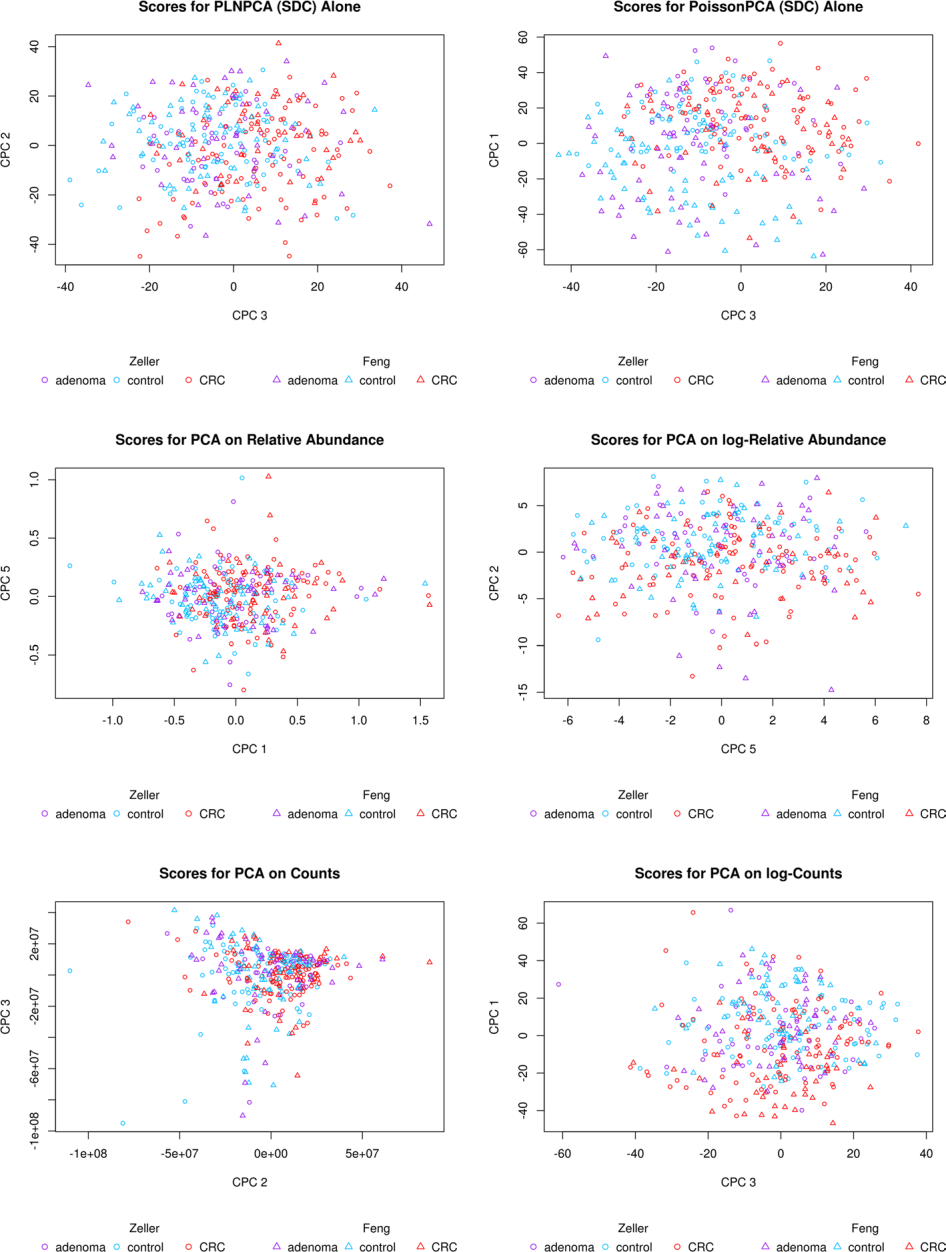
Scores from single-group and naive methods (with SDC for PoissonPCA/PLNPCA) by disease state

Figure 7 depicts selected score plots for each candidate ensemble method with sequencing depth corrections applied (see Additional file 1: Fig. S9 for the equivalent plots with no sequencing depth correction), which show participants with CRC clustering distinctly from participants without CRC on the common axes, with the best performance given by PoissonPCA and SCPCA. This is consistent with the simulations, which provides some evidence that our simulated data are highly similar to real metagenomic data. From Fig. 8, which shows the data projected on several PoissonPCA and SCPCA CPCs for each dataset separately, we can see that the Feng et al. [14] data seem to be better behaved than Zeller et al. [47] in terms of the separation of CRC patients from others. From Figs. 7 and 8, the best clustering of control vs. CRC samples is shown by CPC 4 and CPC 3 (and hence these are the CPCs that we will be analyzing later in order to gain biological insight into the relationships between taxa and disease state). Figure 9 shows the score plots for CPC 1 to CPC 6 by PoissonPCA and SCPCA for both data sets, with the study of origin color-coded: it can be seen that in all CPC directions except CPC 1, study-specific information is not apparent, which is the desired result of a common factor method. The reason that CPC 1 shows some separation between study of origin is mainly because this direction is also a good direction to separate the CRC patients from the control and adenoma patients in the Feng et al. [14] data, as shown in Fig. 8. The fact that there is very little separation by data source suggests that these axes indeed correspond to common, generalizable, CRC-related signal. In contrast, although PoissonPCA alone with SDC on the concatenated data does show clustering by disease state on the selected PCs in Fig. 11, the score plots in Fig. 10 show clustering by study of origin on these and most other PCs. This illustrates how without using a common basis, biological interpretation can become extremely challenging: the single-group PCs that best distinguish disease state in these data also contain signals that are relevant to the exact conditions of one study but are not necessarily relevant to disease state in general, and it will be unknown which taxa are implicated in which. Of the naive PCA methods, each one shows some clustering by disease state (Fig. 11). Figures S10 through S20 show score plots for the rest of the ensemble methods colored by study of origin, with Figures S21 through S25 containing those for the single-group and naive methods. Figures S22 through S25 of the naive methods do not suggest pervasive clustering by study of origin, although this is expected since the data were individually mean-centered by study.

To confirm that the information captured by CPCs does include the crucial signal that distinguishes CRC patients from others, we next investigated the predictive ability of these scores. Since we, like Zeller et al. [47], found that samples positive for colorectal adenoma tended to cluster with control samples, we collapsed the two groups together. As a benchmark of the discriminating signal present in the data, we performed 10-fold CV using random forest models on the full genus-level concatenated data (see Methods for details). We predicted disease state on each test fold, and consider the performance of this powerful nonlinear classifier to represent the realistic upper limit of the extent to which the signal in these data can be used to discriminate CRC samples. This allows us to compare these benchmark results to the classification performances of simple, interpretable generalized linear models on our dimension-reduced CPC scores to determine whether our ensemble method successfully captures relevant biological signal. To this end, we trained and tested logistic regressions on the first five or ten common scores estimated by each method using the same CV folds. The results are summarized in Table 3; entries with mean test-fold values of area under the receiver operating curve (AUC) and accuracy that were not significantly different from the corresponding random forest results are presented in bold.

**Table 3:** Mean test fold AUC and accuracy from 10-fold CV (standard errors in parentheses) for classification of colorectal cancer samples

		First 5 CPCs		First 10 CPCs	
	Scores	AUC	Accuracy	AUC	Accuracy
No SDC	PoissonPCA + SCPCA	0.803 (0.025)	**0.728** (0.022)	0.804 (0.027)	**0.762** (0.022)
	PLNPCA + SCPCA	0.752 (0.033)	0.723 (0.020)	0.751 (0.032)	0.714 (0.025)
	PoissonPCA + FCPCA	0.801 (0.024)	**0.739** (0.022)	**0.809** (0.026)	**0.757** (0.019)
	PLNPCA + FCPCA	0.662 (0.036)	0.677 (0.032)	0.687 (0.042)	0.697 (0.024)
	PoissonPCA + MSFA, q = 4	0.781 (0.027)	**0.725** (0.027)	0.758 (0.028)	**0.725** (0.027)
	PLNPCA + MSFA, q = 2	0.748 (0.032)	0.674 (0.021)	0.750 (0.028)	0.705 (0.022)
	PoissonPCA Alone	0.811 (0.021)	**0.742** (0.022)	**0.817** (0.021)	**0.751** (0.028)
	PLNPCA Alone	0.741 (0.018)	0.700 (0.025)	0.765 (0.021)	0.705 (0.019)
SDC	PoissonPCA + SCPCA	0.806 (0.026)	**0.759** (0.023)	**0.815** (0.026)	**0.771** (0.021)
	PLNPCA + SCPCA	0.773 (0.033)	**0.748** (0.020)	**0.790** (0.033)	**0.751** (0.031)
	PoissonPCA + FCPCA	0.802 (0.025)	**0.754** (0.025)	**0.822** (0.022)	**0.762** (0.016)
	PLNPCA + FCPCA	0.635 (0.028)	0.609 (0.021)	**0.785** (0.031)	**0.756** (0.025)
	PoissonPCA + MSFA, q = 4	0.794 (0.027)	0.742 (0.018)	0.782 (0.030)	**0.748** (0.019)
	PLNPCA + MSFA, q = 3	0.669 (0.027)	0.637 (0.025)	0.701 (0.029)	**0.696** (0.034)
	PoissonPCA alone	0.800 (0.025)	**0.742** (0.025)	**0.822** (0.024)	**0.745** (0.022)
	PLNPCA alone	0.753 (0.019)	0.723 (0.023)	0.768 (0.020)	0.708 (0.017)
Naive	PCA of relative abundance	0.677 (0.015)	0.637 (0.023)	0.670 (0.023)	0.654 (0.024)
	PCA of log relative abundance	0.674 (0.031)	0.638 (0.022)	0.686 (0.031)	0.646 (0.033)
	PCA of counts	0.659 (0.013)	0.637 (0.023)	0.653 (0.020)	0.640 (0.018)
	PCA of log-counts	0.793 (0.027)	**0.719** (0.020)	0.798 (0.026)	**0.756** (0.02)
		**AUC**	**Accuracy**		
	Random forest (all features)	0.853 (0.019)	0.773 (0.012)		

First, the naive methods did not perform optimally, although like in the simulation, PCA on the log-counts works the best among them. In general, the ensemble methods perform well, with many obtaining high accuracy using only five CPCs, although ten CPCs provide even better results. In fact, the linear classifier using just 5–10 ensemble method scores as predictors often performed as well (at the 5% significance level) as the random forest classifier using all genus-level taxa, which suggests that the log transformation used in PoissonPCA and PLNPCA is suitable for microbial abundance data, and that virtually all of the important shared signal characterizing CRC samples was successfully captured during dimension reduction. Also, almost all of the ensemble methods work slightly better (although mostly non-significant at the 5% level) with SDC than without, which shows that sequencing depth correction is important in real data analysis.

The results also show that methods using PoissonPCA’s variance estimator consistently perform better in prediction than those employing PLNPCA, regardless of whether sequencing depth correction is applied and regardless of which common factor method is applied. The best-performing methods were PoissonPCA with FCPCA, and PoissonPCA with SCPCA, so our recommendation is to use PoissonPCA with SCPCA because SCPCA is robust to slight indefiniteness in $\Sigma _s$. MSFA was tested using *q* values from 1 to 5, and performed fairly well with PoissonPCA, but for $q=4$ and $q=5$ MSFA was either very slow to converge (using PoissonPCA) or failed to converge at all (using PLNPCA).

PoissonPCA alone on the concatenated data also performed well. Figure 8 (and the fact that the Zeller dataset contains more observations than Feng) suggests that the ability to discriminate disease state in Zeller samples may be the biggest determinant of classification performance, and so the success of PoissonPCA alone on the concatenated data may be due to some unique biological signal that is relevant to CRC status in the Zeller data but missing or undetectable in the Feng data, which would naturally give the single-group procedures an edge over the ensemble methods for prediction. In general, unique signal could be helpful or unhelpful for predicting a given response, but in either case it could potentially obstruct meaningful interpretation of the predictors. The results in Table 3 support our hypothesis that the proposed ensemble methods—especially those that use PoissonPCA followed by a CPCA approach—are able to find a low-dimensional representation of the data that retains virtually all of the discriminating biological signal that is shared among groups, which is our main interest.

**Fig. 12:**
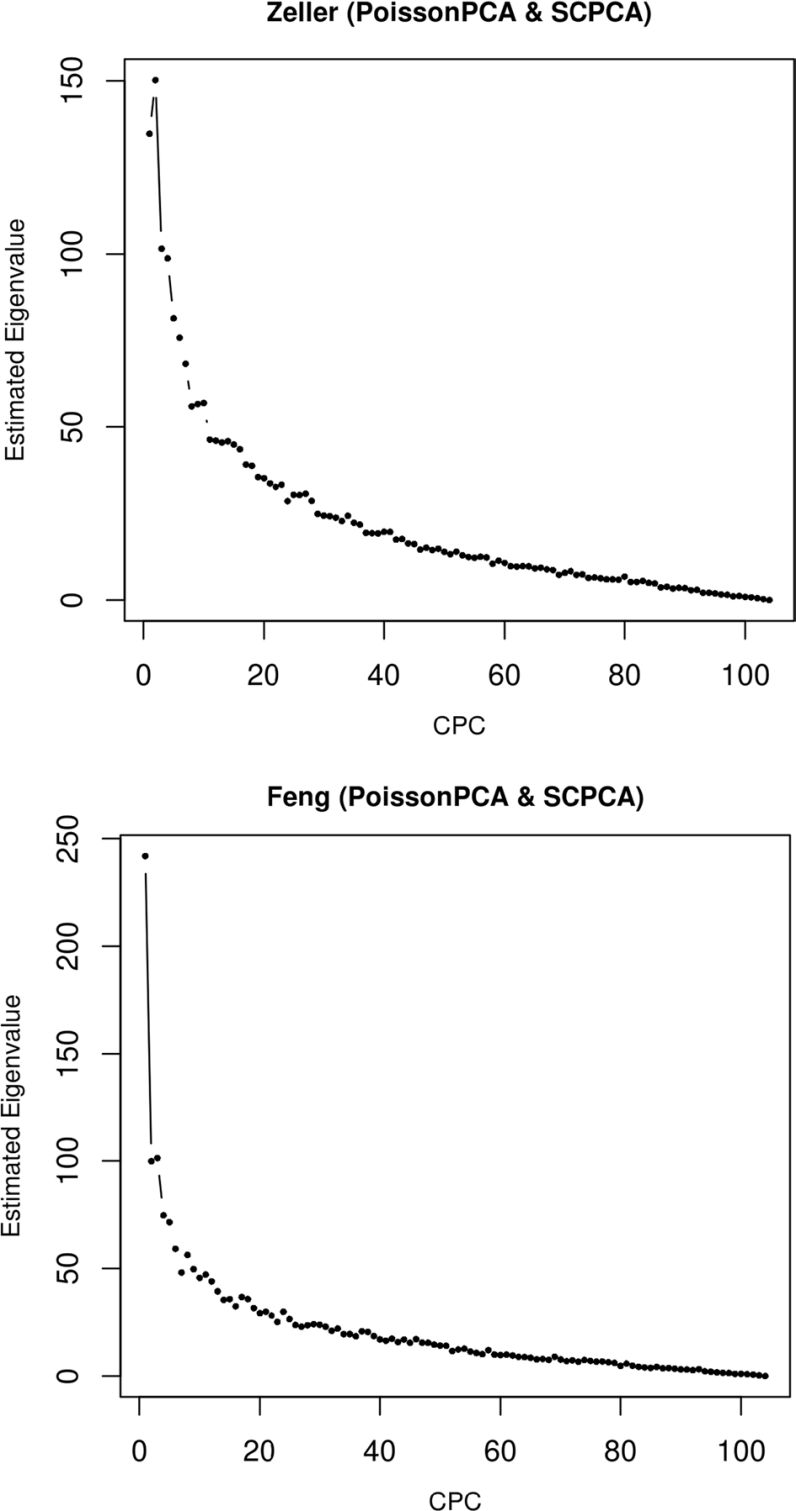
Scree plots of PoissonPCA followed by SCPCA on the Zeller and Feng data

**Fig. 13:**
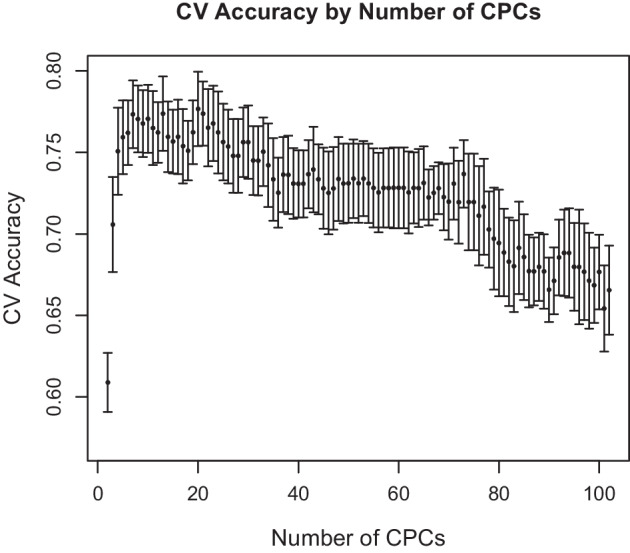
Cross-validation accuracy of logistic regression models by number of CPC scores used (scores obtained from PoissonPCA and SCPCA on the Zeller and Feng datasets)

To choose the optimal number of common principal components to describe the common signal on CRC status, we can choose *q* using scree plots of the estimated eigenvalues or by prediction such that the average test fold misclassification error is lowest. For example, using PoissonPCA followed by SCPCA, scree plots in Fig. 12 suggest that the Feng data have a pronounced elbow, while the eigenvalues level off more slowly in the Zeller data. However, in both datasets, the last large difference between successive eigenvalues occurs by about $q=11$. In Fig. 13, which shows the cross-validation accuracies, it appears that the classification accuracy reaches near its optimum around 10 CPCs and tends to decrease from that point on, so we might choose $q=10$. The prediction results in Table 3 corroborate that in general, such parsimonious models perform well.

Finally, we turn to biological interpretation of the common loadings, wherein lies the strength of the ensemble method. Even assuming that single-group methods were able to resolve some common underlying variation patterns, the resultant PCs reflecting this would be indistinguishable from those capturing within-study variation or contrasts between studies. However, we have been assured by our simulation results and assessment of the CPC scores that the ensemble methods can yield $q<<p$ common axes that load on fully shared factors with strong signal. Furthermore, since our estimated loadings are related to the decomposition of variance on the log scale, we can interpret the values given by these loadings as log ratios of geometric means between groups of taxa with large-magnitude values in opposite directions. The following was observed from the common loadings estimated from SCPCA on the PoissonPCA sequencing depth-corrected variance estimate.

Within our two CRC datasets, we found that on the two estimated common loading vectors that appeared to differentiate between samples with and without CRC, taxa with loading coefficient magnitudes in the 90th or greater percentile were often described in other studies of CRC-associated microbiota. Peptostreptococcus, Butyrivibrio, Phascolarctobacterium, Fusobacterium, and Porphorymonas genera were identified by our common loadings as well as by the analyses of Zeller et al. [47], while Acidaminococcus, Parvimonas, Gemella, and Peptostreptococcus were found in common with Feng et al. [14]. Genera we identified that have been previously associated with CRC in additional studies include Fusobacterium [10, 36], Porphorymonas [10, 36], Acidaminococcus [3], Phascolarctobacterium [44], Enterococcus [23], Gemella [23], Klebsiella [10], Prevotella [36], Solobacterium [46], and the Siphoviridae viruses [19]. Of those highlighted by our analyses, only Dialister [44], Butyrivibrio [47], and Flavonifractor [1] have been reported to be protective. Lastly, our loadings suggest that Adlercreutzia contributes to distinguishing CRC samples, but this genus has not yet been implicated in the literature, although it has been observed to participate in the metabolism of flavonoids [27], which are antioxidants generally known to be anti-inflammatory and anti-tumorigenic. Moreover, on the loading in which Butyrivibrio (a genus of butyrate producers) and Adlercreutzia have large negative coefficients, all other large magnitude coefficients are positive. Flavonoids, along with dietary fibre, are linked to butyrate production, all of which have been linked to metabolic health and decreased risk of cancer [20, 32, 45], although little is known about how flavonoids are metabolized. This loading vector may then represent a contrast between these genera—which may be involved in some protective pathway related to the metabolic intermediates of flavonoids—and the other genera dominating the loading. This is an insight that neither Zeller et al. [47] nor Feng et al. [14] were able to resolve from their single-group analyses.

## Conclusion

We have demonstrated that by appropriate modeling of microbial abundance count datasets so as to estimate their respective variances first, followed by estimation of a basis for a common low-dimensional subspace for the true underlying abundances, we can remove unwanted variation while retaining shared variation patterns. Through simulation studies and real data analysis, we can make a recommendation on the best ensemble method among 12 ensemble methods: PoissonPCA (with sequencing depth correction) and SCPCA ensemble performs the best. The similarity of the simulated data to the real data provides a basis for us to make this general conclusion that the proposed ensemble method will be applicable to real microbial abundance data, which is further confirmed by our real data analysis.

While we have demonstrated the method on data from two studies, it can be readily applied to any number of datasets. PoissonPCA and SCPCA, the constituent methods showing the best performance, computation speed, and robustness, can scale up to handle estimation of a common basis for many groups. Because of the abundance of biomedical and clinical data available from studies of human and animal gut microbiota that tend to investigate similar themes, our method already has a solid basis of applicability based on our results. In addition, since taxonomic abundance data from 16S sequencing and metagenomic sequencing each pose similar obstacles to classical statistical analyses, the exploratory methods we propose apply to both.

Since the proposed method is only a data exploration tool, like other exploratory analysis methods it relies on further interpretation, modelling, or experiments to judge whether the common directions found represent biologically meaningful signal. In reality, it is possible that the “common factors” found could be due to either biological or technical common signals, or just a mathematically optimized direction without clear meaning. To support our simulation study findings that the proposed ensemble method can faithfully reconstruct the true shared variance, in the real data analysis example we were able to accurately classify metagenomic samples based on a logistic regression model using 5–10 CPC scores, which confirmed that the discrimination signal is contained in the information captured by the CPC scores. We established this by comparing the results of logistic regression on 5–10 scores to the results from a state-of-the-art machine learning method trained on all taxa, since the latter provides an indication of the realistic classification accuracy limit of the dataset. Because the linear classifiers trained only on the first several scores performed as well as a far more flexible method on the full data, we were able to conclude that the CPC directions and scores contain almost all relevant signal that distinguishes CRC patients from normal controls. This in turn suggests that our analysis of the loadings is likely to implicate taxa that reliably distinguish disease state across studies. With more groups of data obtained under different technical settings, under the condition that there should be some common true biological signal hidden in all groups of data sets, shared large variance more likely represents biological signals. As a data exploration tool, the method can also be used for data visualization and provides some insight for further statistical modelling. For example, we can decide if linear methods or nonlinear methods are needed based on the principal scores or the reconstructed log-scale microbial abundance data in a regression or classification problem.

It is also straightforward to extend our method to detect both common and unique signals among different microbial abundance data sets. This can be realized by two steps: the first step is to run our ensemble method to find *q* and compute the first *q* CPCs; then in the second step, by subtracting the covariance extracted by the CPCs from the estimated covariance matrix for each group, we obtain for each group a covariance matrix spanning the space orthogonal to the space spanned by the CPCs, and separately eigen-decomposing each of these resultant matrices can extract the large unique biological or technical variation. In this way, the scope of our method could be extended to exploring commonalities in community structure that are conserved across a diverse range of communities.

In conclusion, the study of microbiota poses a number of challenges, including heterogeneous signal-obstructing noise. In this paper, we addressed the lack of methods for cross-study generalization in microbial abundance data, and proposed a framework to “remove” some of the observed within-study variation by seeking the latent structure that provides a scaffold for the common variance. We found that our two-step ensemble approach—first estimating the variance of the underlying abundances on the log scale for each group by PoissonPCA, and then decomposing the variances with a multi-group method (SCPCA)—faithfully captured shared signal in our simulation scenarios, even when the groups had significant unique signal. The analysis of the Feng and Zeller colorectal cancer datasets demonstrated the excellent dimension reduction and signal retention capabilities of the ensemble methods, and moreover the ease with which biological interpretations can be drawn from the common loadings.

## Methods

We will operate under the assumption that signal shared among *S* different datasets on the same *p* variables is likely to represent biological variability of interest, whereas disparate signal likely reflects variability attributable only to irrelevant differences in experimental conditions. We propose using a two-step ensemble method, with the first step obtaining estimated variance-covariance matrices for each dataset by PoissonPCA or PLNPCA, and the second step to simultaneously decompose them to find a *q*-dimensional basis that is common to all groups. For the second step, we compare two general approaches: one possible approach is a multi-group extension of PCA called common principal components analysis (CPCA), first described by Flury [15], which assumes that there exists an orthogonal matrix that can approximately diagonalize the covariance matrices of all *S* groups simultaneously. We test two algorithms for CPCA: Flury’s CPCA (FCPCA) and stepwise CPCA (SCPCA) [42]. The other model under consideration is the previously mentioned MSFA [12], which is an extension of classical factor analysis. Both CPCA and MSFA require Wishart or multivariate normal assumptions, which our variance estimates approximately satisfy for real microbiome data due to the log transformation. After estimating the common loadings, the final step of our proposed ensemble method is to express the underlying abundances in each multivariate observation with respect to our new common basis, which we will do by projecting the estimated latent Poisson means from each group into a common subspace spanned by the loadings using a quasi-likelihood procedure that Kenney et al. [22] developed for the single-group case.

To provide insight into why the ensemble method can eliminate most unshared technical noise while retaining shared biological signal and facilitating novel exploratory findings, we will review the main ideas of each method first and then introduce our simulation schemes.

### Estimation of variance

Before we can estimate the common signal among several datasets, we first have to deal with the Poisson error in each dataset individually. In this work we will consider two approaches, each of which assumes that the observations are conditionally Poisson-distributed. Since our application is the analysis of microbiome composition, we are primarily interested in treating the means on the logarithmic scale, which arises naturally in the first method via the canonical link function, and in the second method can be achieved by a transformation.

#### Poisson log-normal PCA

The Poisson log-normal PCA (PLNPCA) [11] is a fully parametric model that extends the probabilistic PCA of Tipping and Bishop [40] such that the emission layer is Poisson (or any natural exponential family distribution) rather than normal. Let $X_i \in \mathbb {N}^p$, $i=1,\dots ,n$, be a random vector of which we have observed *n* realizations. PLNPCA assumes that the $X_i$ are conditionally independently Poisson-distributed given the log Poisson means $\log \Lambda _i$, where $\log$ is assumed to be applied element-wise. In the parameter space dwells $\log \Lambda _i\in \mathbb {R}^p$, $\log \Lambda _{i} = \xi _i + \mu + \beta w_{i}$ for $i=1,\dots ,n$, where $w_{i}$ are iid $\mathcal {N}(0_{\ell },I_{\ell })$. That is, $\log \Lambda _{i}$ is a latent variable and follows a multivariate normal distribution with mean $\xi _i + \mu$ and variance $\Sigma =\beta \beta ^T\in \mathbb {R}^{p \times p}$. No uniqueness constraints are put on $\beta$. Using this framework one has the option of introducing row-wise sums as an offset to treat sequencing depth as observed sampling effort by $\xi _i\in \mathbb {R}^p$ (i.e., $\xi _{i1}=\xi _{i2}=\dots =\xi _{ip}$). In the microbiome setting, we choose these offsets to correspond to log-total read count, which is the closest we have to an observed value for sequencing depth. Should we wish not to apply a sequencing depth correction, the following results hold as written for $\xi _i=0_p$.

Because the marginal likelihood isn’t analytic for the Poisson case, the authors integrate out *w* under a variational approximation of *p*(*w*|*X*), and maximize the variational lower bound for the marginal log-density of *X* instead of maximizing the marginal log-likelihood. An estimate of the variance-covariance matrix of the log means ($\Sigma =\text {Var}(\log \Lambda )$) can be obtained using variational inference.

PLNPCA also provides the opportunity to estimate a low-rank covariance matrix depending on the dimension chosen for the latent space, but for our ensemble method we wish only to reduce dimension based on the common variation across all *S* datasets, so we will be using the rank-*p* estimate. Also, we wish to avoid disrupting signal in a supervised fashion so we will not include any covariate effects in the model. Details of this process and the resultant variance estimator can be found in their paper.

PLNPCA is implemented in the R package **PLNmodels** [11].

#### PoissonPCA

PoissonPCA [22] also assumes that conditional on the latent Poisson means $\Lambda _{ij}$, the $X_{ij}$ are independently distributed as Poisson with parameter $\Lambda _{ij}$. In contrast to PLNPCA, the distribution of $\Lambda$ itself is not parameterized. The authors derived an unbiased variance estimate using method of moments for any nonlinear transformation of the latent means, in particular for estimating $\Sigma =\text {Var}(\log \Lambda )$, which we use in this paper.

The main idea of PoissonPCA is to look for an element-wise transformation *f* for the data, *f*(*X*), such that conditionally on $\Lambda$, the mean of *f*(*X*) is $\log \Lambda$. Accordingly, substituting $\log \Lambda$ for $\mathbb {E}[f(X)|\Lambda ]$ in the total variance $\text {Var}(f(X))=\mathbb {E}[\text {Var}(f(X)|\Lambda )]+\text {Var}(\mathbb {E}[f(X)|\Lambda ])$, we get

$$\begin{aligned} \Sigma =\text {Var}(\log \Lambda )=\text {Var}(f(X))-\mathbb {E}[\text {Var}(f(X)|\Lambda )] \end{aligned}$$

Estimating the conditional variance $\text {Var}(f(X)|\Lambda )$ is achieved in PoissonPCA by finding a function *k*(*X*) such that the average conditional mean of *k*(*X*) is approximately $\text {Var}(\log X|\Lambda )$.

PoissonPCA accounts for sequencing depth by introducing a random variable $\xi _i\in \mathbb {R}^p$ such that $X_{ij}|(\Lambda _{ij},\xi _{ij}) \sim \text {Poisson}(\xi _{ij}\Lambda _{ij})$, where $X_{i1},\dots ,X_{ip}$ are independent given $\Lambda _i$ and $\xi _i$. Unlike in PLNPCA, $\xi _i$ is not considered to be observable, so under this model we end up estimating $\Sigma =\text {Var}(\log \Lambda )=\text {Var}(\log (\xi \circ \Lambda ^*))$, where $\circ$ is the element-wise product, when in fact what we want is $\Sigma ^*=\text {Var}(\log \Lambda ^*)$. Thus, in order to account for sequencing depth error, PoissonPCA adds constraints to the variance estimator given above in order to get the desired estimator $\Sigma ^*$. Two methods were developed in [22] for sequencing depth correction. The one we implement in the present study, the compositional correction, assumes that $\Sigma ^*$ should be symmetric and contained in the orthogonal complement of the vector 1, which leads to the sequencing depth-corrected variance estimate as

$$\begin{aligned} \Sigma ^*=\Sigma -(pI_p+1_p1_p^T)^{-1} \Sigma 1_p1_p^T - 1_p1_p^T\Sigma (pI_p+1_p1_p^T)^{-1}. \end{aligned}$$

They also developed a semi-parametric method for estimating the scores of the transformed means, which we adapt for use in our ensemble method.

Note that the construction of these estimators makes no guarantee about the definiteness of the matrices, and in practice there can be several negative terminal eigenvalues. Since we found that the multi-group methods FCPCA and MSFA (to be described in the following sections) were sensitive to whether or not input variance-covariance matrices were positive-definite, if any $\hat{\Sigma }_s$, $s=1,\dots ,S$ computed from PoissonPCA in our analyses was indefinite, we eigen-decomposed it, replaced the negative eigenvalues with small decreasing positive values, and then used the eigenvectors to reconstruct the variance before running FCPCA or MSFA.

PoissonPCA is implemented in the R package **PoissonPCA** [22].

### Multi-group analysis

We are ultimately interested in estimating the loadings that are common to all groups, and projecting the estimated latent Poisson means from each group into a common space spanned by the loadings. To achieve this, the natural choice would be to apply multi-group extensions of PCA or of factor analysis to the Poisson-corrected estimates from each group, since by dealing with abundance on the log scale we are able to decompose our variance estimates under Wishart/multivariate normal assumptions. Both PCA-based and factor analysis-based approaches allow us to find a common space of low dimension, but the latter is prescriptive in this sense while the former inherits the exploratory nature of PCA.

#### Common principal components analysis

Perhaps the most direct multi-group generalization of PCA is Flury’s common principal components (FCPCA) [15], which assumes that there exists an orthogonal matrix that can approximately diagonalize the covariance matrices of all *S* groups simultaneously. Using a generalized PCA approach, the dimension of the common space can be chosen after estimating the full loadings matrix, based on which *q* loading vectors are associated with the largest variances for all groups simultaneously. Unlike the usual PCA, FCPCA assumes that the sample covariance matrices follow a Wishart distribution, and so the common loadings matrix is estimated in a maximum likelihood framework.

Let $\Sigma _s \in \mathbb {R}^{p \times p}$ be symmetric and positive definite and assume that each $(n_s-1) \hat{\Sigma }_s$ is independently distributed as $\textrm{W}_p(n_s-1, \ \Sigma _s)$, $s=1,\dots S$, where $\textrm{W}_p$ is the *p*-variate Wishart distribution. If there is a rotation matrix $V \in O(p)$ (where *O*(*p*) denotes the set of orthogonal $p \times p$ matrices) for which

1 $$\begin{aligned} V^T \Sigma _s V = D_s \end{aligned}$$

for all *s*, where $D_s={{\,\textrm{diag}\,}}(d_{s1},\dots ,d_{sp})$, then the subspace spanned by the columns of *V* is common to all groups; the assumption in FCPCA is that *V* exists as such. Hence, FCPCA comes down to simultaneous diagonalization (or an approximation thereof) of the *S* sample covariance matrices. Fortunately, for $\hat{\Sigma }_1,\dots ,\hat{\Sigma }_S$ positive-definite, their quadratic forms are strictly convex, and so the optimization problem posed in FCPCA is highly tractable; see [16] for details of the algorithm. Using FCPCA we can reduce the basis to $v_1,\dots ,v_q$ only provided that the last $p-q$ eigenvalues are small for all *S* groups.

More recently Flury’s algorithm was revisited by Trendafilov [42], who criticized the fact that FCPCA does not constrain the eigenvalues of each group to be simultaneously decreasing, which in some cases could disallow its use as a dimension reduction strategy. Instead, Trendafilov [42] suggested the stepwise algorithm (which we will call SCPCA), which finds the common loadings sequentially in order of variance explained. SCPCA [42] starts with the same objective function as FCPCA, but performs minimization to find the optimal axes sequentially based on the fact that if covariance matrices $C_1,\dots , C_S$ share a common eigenvector *e*, then *e* is also an eigenvector of the average of $C_1,\dots ,C_S$ weighted by their unique eigenvalues associated with *e*. If the eigenvalues estimated by FCPCA for each estimated covariance matrix are all simultaneously decreasing, then it can be shown that SCPCA yields the same result as FCPCA. However, if the FCPCA eigenvalues are not simultaneously decreasing in all *S* groups, then the stepwise approach will not solve the minimization problem. Trendafilov [42] argues that despite this, the stepwise solution is useful because it can always be used to find a set of $q \le p$ common principal component vectors forming a basis for $\mathbb {R}^q$, such that their variances $d_{s1},\dots , d_{sq}$ are approximately simultaneously decreasing for all $s=1,\dots ,S$, and all $d_{1j},\dots ,d_{Sj}$ are as similar as possible for a given *j*, $j=1,\dots ,q$.

In the present study, we compared the performance of FCPCA and SCPCA for simultaneously decomposing the *S* covariance matrices that have been estimated using either PoissonPCA or PLNPCA. Our FCPCA and SCPCA were implemented using code adapted and modified from source code in the R packages **multigroup** and **cpca** respectively.

#### Multi-study factor analysis

Multi-Study Factor Analysis (MSFA) [12] is an extension of classical factor analysis and likewise assumes that the latent variables and measurement error are multivariate normal and hence that the observations have a multivariate normal marginal distribution. However, in MSFA, there are *q* latent common factors and $\ell _1,\dots ,\ell _S$ latent unique factors, and so $S+1$ loadings matrices have to be estimated by maximum likelihood, which is a much more difficult optimization problem than that posed by classical factor analysis. Also, as with any factor analytic approach, dimensions of the latent subspaces must be considered a hyperparameter of the generative model.

Let $X_{is}\in \mathbb {R}^p$, $i=1,\dots ,n_s$, $s=1,\dots ,S$ be a random vector with $n_s$ realizations in the $s^\text {th}$ group. MSFA assumes that there exist iid latent variables $f_{is} \in \mathbb {R}^q$ and $w_{is} \in \mathbb {R}^{\ell _s}$, which generate $X_{is}$ by

$$\begin{aligned} X_{is}=\Phi f_{is}+\beta _s w_{is}+\mu _s+\epsilon _{is}, \ \ \text {with} \ \ f_{is}\sim \mathcal {N}(0_q,\text {I}_q), \ w_{is}\sim \mathcal {N}(0_{\ell _s},\text {I}_{\ell _s}) \end{aligned}$$

for $i = 1, \dots , n_s$, $s=1, \dots , S$, where $\mu _s\in \mathbb {R}^p$ is the mean vector, $\epsilon _{is}$ are iid with $\epsilon _{is}\sim \mathcal {N}(0_p,\Psi _s)$, where $\Psi _s={{\,\textrm{diag}\,}}(\psi _{s1},\dots ,\psi _{sp})$, and $\epsilon _{is}$ is independent from the latent factors $f_{is}$ and $w_{is}$. $\Phi$ is a $p \times q$ matrix of common loadings, and $\beta _s$ is a $p \times \ell _s$ matrix of group-specific loadings, which provide structure to the latent factors. Alternatively, we can say that each $X_{is}$ is conditionally independent given the latent variables $f_{is}$ and $w_{is}$ as follows:

$$\begin{aligned} X_{is}|f_{is},w_{is} \sim \mathcal {N}(\Phi f_{is}+\beta _s w_{is} + \mu _s, \Psi _s) \ \ \text {with} \ \ f_{is}\sim \mathcal {N}(0_q,\text {I}_q), \ w_{is}\sim \mathcal {N}(0_{\ell _s},\text {I}_{\ell _s}). \end{aligned}$$

Since there is a multivariate normal distribution for $X_{is}$ conditional on the multivariate normal latent variables, $X_{is}$ has a multivariate normal marginal distribution $X_{is} \sim \mathcal {N}(\mu _s,\Sigma _s=\Phi \Phi ^T + \beta _s \beta _s^T + \Psi _s)$. Hence, the log-likelihood is given by

$$\begin{aligned} \mathcal {L}(\Phi , \beta _s, \Psi _s)&=\log \prod _{s=1}^S \prod _{i=1}^{n_s} p(X_{is}|\Phi , \beta _s,\Psi _s)\\&\propto \sum _{s=1}^S \big (-\frac{n_s}{2} \log |\Sigma _s|-\frac{n_s}{2} \textrm{tr} (\Sigma _s^{-1}\hat{\Sigma }_s)\big ). \end{aligned}$$

From this we see that even though MSFA is designed for normally distributed data, we can use it as the multi-group step in our ensemble method because its likelihood depends only on the estimated variance-covariance matrices for each group.

As identifiability constraints, the authors impose that $\Phi$, $\beta _1, \dots , \beta _S$ all be lower triangular matrices, which is typical of classical factor analysis and forces the first loading to correspond only to the first axis of the factor space, the second loading to the first and second axes, and so on and so forth. The authors further note that an additional constraint—that $\text {rank}\big ([\Phi \ \beta _1 \dots \beta _S]\big )=q+\sum ^S_{s=1}\ell _s$—is needed to ensure uniqueness of the solution, since we have to estimate *S* group-specific loadings plus the common loadings from the *S* covariance matrices. $\hat{\Phi }$, $\hat{\beta }_1,\dots ,\hat{\beta }_S$, and $\hat{\Psi }_s$ are estimated by expectation-conditional maximization (ECM). Of course, we are interested only in $\hat{\Phi }$, the matrix that describes how the latent factors characterizing the common signal are weighted to generate the observed data.

MSFA was implemented using the source code from the R package **msfa** [12], modified so as to use $\hat{\Sigma }_1,\dots ,\hat{\Sigma }_S$ estimated from PoissonPCA/PLNPCA instead of from the standard sample covariance matrices. Additionally, for our method we require a subspace spanned by a set of orthonormal vectors in order of the variance on the common factors, and so what we seek are actually the rotated loadings $v_1,\dots ,v_q$, or the first *q* columns of $V \in O(p)$ computed from $\hat{\Phi }\hat{\Phi }^T$ by the following spectral decomposition:

2 $$\begin{aligned} \hat{\Phi }\hat{\Phi }^T=V A V^T, \end{aligned}$$

where *A* is the diagonal matrix of eigenvalues.

#### Computing scores

Finally, after estimating the common loadings with either SCPCA, FCPCA, or MSFA, we want to express the underlying abundances in each sample with respect to our new common basis, and we will refer to these quantities as the scores.

Since, in all cases, for any given multivariate observation we are interested in the scores of the unobserved $\log \Lambda _{is}$ rather than the scores of the observed $X_{is}$, we adopt the following procedure from Kenney et al. [22]. To apply the classical PCA criterion of minimizing the squared reconstruction error between the original points and their projections onto principal component space, we minimize the squared reconstruction error between $\log \Lambda _{is}$ and its projection $P_{is}^q$ onto the *q*-dimensional common subspace spanned by the orthonormal vectors $v_1,\dots ,v_q$ that we estimated using CPCA or MSFA. Since $\Sigma _s$ is the variance of $\log \Lambda _s$, this error is given by the squared distance

3 $$\begin{aligned} D^2=(\log \Lambda _{is}-\mu _s-P_{is}^q)^T\Sigma _s^{-1}(\log \Lambda _{is}-\mu _s-P_{is}^q), \end{aligned}$$

where, because the $v_j$’s are orthonormal, the projection is $P_{is}^q=\sum ^q_{j=1}v_jv_j^T(\log \Lambda _{is}-\mu _s)$. At the same time, since $\log \Lambda _{is}$ is unobserved, we should still seek to maximize the likelihood of the observed data. Whether we use PoissonPCA or PLNPCA to estimate $\Sigma _s=\text {Var}{(\log \Lambda )}$, the underlying assumption is always that $X_{ijs}$ was generated by a Poisson distribution with mean $\Lambda _{ijs}$. So as in the single-group case in Kenney et al. [22], by combining the Poisson log-likelihood and equation (3), we arrive at an objective function of the form

$$\begin{aligned} L(\Lambda _{is})=\sum ^{n_s}_{i=1}\big (X_{is}^T\log \Lambda _{is}-1^T\Lambda _{is}-(\log \Lambda _{is}- \mu _s-P_{is}^q)^T\Sigma _s^{-1}(\log \Lambda _{is}-\mu _s-P_{is}^q)\big ). \end{aligned}$$

This is optimized by Newton–Raphson iteration in the **PoissonPCA** R package, and we implement the procedure using adapted portions of this code.

### Ensemble method

We have reviewed two very different ways of estimating the full- (or near full-) rank variance-covariance matrix from a set of conditionally independent realizations of Poisson sampling in which the Poisson means are subject to additional multiplicative noise: PoissonPCA and PLNPCA, each of which can either be performed with a sequencing depth correction or without. These methods are applied to each data set $X_{1},\dots ,X_{S}$ individually. We went on to explore two distinct methods that can take a set of *S* estimated variance-covariance matrices and estimate a set of $q<p$ common vectors forming a shared orthogonal basis for a common *q*-dimensional subspace of $\mathbb {R}^p$: CPCA (for which we have a choice of two algorithms, SCPCA and FCPCA) and MSFA. The twelve possible combinations of variance estimation and common factor extraction techniques are given in Table 1.

### Simulation study

We performed simulation studies of two synthetic groups of multivariate Poisson log-normal observations across several scenarios. These scenarios differed on the true signal (including the number of eigenvectors that were common to both groups’ variance-covariance matrices, and whether the eigenvalues of the variance-covariance matrices were simultaneously decreasing), the sample sizes $n_1$ and $n_2$, whether or not the sample sizes were balanced, and whether or not sequencing depth correction was performed in the variance estimation stage. For each simulation experiment, the process of simulating Poisson log-normal data and applying each method was performed 100 times.

For each of the 100 replicates, synthetic data for two “groups” were simulated as follows. Synthetic eigenvectors $E_1$ were constructed for Group 1 by spectral decomposition of a synthetic covariance matrix $FF^T$ where each column $F_j$, $j=1,\dots ,p$ was a normalized length-*p* vector of standard normal variates. We then constructed the eigenvectors $E_2$ for Group 2 so as to share the first *q* columns of $E_1$ for several values of *q*, while the remaining columns $e_{2,q+1},\dots ,e_{2,p}$ were replaced by the normalized residuals of vectors of standard normal variates regressed on the preceding *q* columns. These eigenvectors, along with a pre-determined set of eigenvalues for each group, were used to construct variance-covariance matrices $\Sigma _1$ and $\Sigma _2$. Note that with decreasing eigenvalues, for $q<<p$, by this construction each shared eigenvector will be a principal eigenvector of the two covariance matrices, whereas with non-decreasing eigenvalues some of the large variances will not be associated with the shared axes: we performed simulation experiments for both these scenarios. Next, these covariance matrices $\Sigma _1$ and $\Sigma _2$ in turn were used to simulate the transformed latent Poisson means $\log \Lambda _1$ and $\log \Lambda _2$ using the multivariate normal, with mean vectors for each group consisting of *p* normal random variates (see Table 4) such that the means differed between simulation replicates only. We then performed scalar multiplication of $\Lambda _1$ and $\Lambda _2$ respectively by length-$n_1$ and length-$n_2$ vectors of gamma random variates to simulate sequencing depth error, and finally these means were used to generate $n_1 \times p$ and $n_2 \times p$ synthetic data matrices of Poisson random variates for each group respectively (see Table 4). Note that since the columns of $\Lambda _1$ and $\Lambda _2$ are all samples from log-normal distributions, they are mostly long-tailed. Because the Poisson means vary between samples, the marginal distributions of counts are both over-dispersed and sparse to a similar extent to real data. Detailed comparisons between the simulated data and the real data are given in Section 7.3 and Supplementary Appendix F.4 of Kenney et al. [22].

**Table 4:** Distributions used to simulate Poisson log-normal data

	Group 1	Group 2
$\log \Lambda _{is}\sim \mathcal {N}(\mu _{is},\Sigma _s)$	$\mu _{ij1}\sim \mathcal {N}(4, 3^2)$	$\mu _{ij2}\sim \mathcal {N}(3,2^2)$
$X_{ijs}\sim \text {Poisson}(\gamma _{is}\Lambda _{ijs})$	$\gamma _{i1}\sim \text {Gamma}(7, 1)$	$\gamma _{i2}\sim \text {Gamma}(10, 1)$

Then, the candidate ensemble methods listed in Table 1 and some single-group alternatives were performed on the synthetic data. The single-group methods were all run on the data from the two groups concatenated together, and these methods comprised PoissonPCA, PLNPCA, naive PCA on counts, naive PCA on log counts, naive PCA on relative abundances, and naive PCA on log relative abundances. Before log-transforming count or relative abundance data for naive PCA, zero values were first imputed with 0.001.

In the case of SCPCA and FCPCA, the explained variances (eigenvalues) for each estimated orthogonal loading vector were computed by

4 $$\begin{aligned} \hat{d}_{sj}= \hat{v_j}^T \Sigma _s \hat{v_j}, \ j=1,\dots ,p, \ s=1,2 \end{aligned}$$

where $\Sigma _s$ is the true variance-covariance matrix of $\log \Lambda _s$. Cumulative sums of $\hat{d}_{11},\dots ,\hat{d}_{1p}$ and $\hat{d}_{21},\dots ,\hat{d}_{2p}$ were divided by the true eigenvalues $\sum ^p_{j=1}d_{1j}$ and $\sum ^p_{j=1}d_{2j}$ respectively to find the proportion of the true variance explained by each method.

In the case of MSFA, as the common loadings are not constrained to orthogonality, the variance $\hat{\Phi }\hat{\Phi }^T$ of the common factors was computed and then eigen-decomposed as described in equation (2). The resultant $\hat{v}_1,\dots ,\hat{v}_p$ (of which only the first *q* contain signal, but all *p* are retained in this case for ease of visibility in plots) were used to compute the estimated eigenvalues and the proportion of true variance explained as according to equation (4).

### Real data analysis

Zeller et al. [47] and Feng et al. [14] collected fecal samples and processed them as described in their respective publications. Although each team had their own bioinformatic pipeline to process the raw reads, the taxonomy tables used for our data analysis were obtained from the R package **curatedMetagenomicData** [35], whose authors applied a standard pipeline for assembly, gene prediction, and taxonomic assignment to the raw files from each study.

The taxonomic abundance tables for each dataset included strain-level taxa from all three domains of life, as well as viruses. Since our candidate methods do not involve any regularization, we separately collapsed the data to the genus level and removed features with near-zero variance to reduce the number of taxa to $p=104 < \min (n_Z=199,n_F=154)$. We then subsetted the features to include only those common to the two datasets, and ran the twelve candidate combinations of methods listed in Table 1 just as we did for the simulation experiments. We then computed the scores by projecting the latent means into the common space.

For disease state prediction, we collapsed colorectal adenoma labels together with control labels. We used the R package **caret** for data splitting and model training. As a benchmark of the discriminating signal in the data, we trained and tested random forest models (using the method implemented in the R package **randomForest**) on the full genus-level concatenated data using 10-fold cross-validation (with folds stratified by disease state). We fit 5000 trees and tuned the hyperparameter mtry with internal 10-fold CV based on classification accuracy. We predicted disease state for each test fold and computed mean AUC and mean accuracy. We consider the performance of this nonlinear classifier to represent the extent to which the signal in these data can be used to discriminate CRC samples. We then trained and tested logistic regressions on the first 5 or 10 common scores for each exploratory method using 10-fold CV (using the same folds as for random forest) and similarly computed mean AUC and accuracy over the test folds.

### Supplementary Information

**Additional file 1. **Supplemental Figures for simulation and real data analysis results.

## Data Availability

The Feng and Zeller datasets are available in the R package curatedMetagenomicData [35], which can be downloaded from https://bioconductor.org/packages/curatedMetagenomicData/.
