# Supplementary material for: Cross-study analyses of microbial abundance using generalized common factor methods
Source: BMC Bioinformatics. 2023 Oct 9;24:380. doi: 10.1186/s12859-023-05509-4 (PMC10561484; doi:10.1186/s12859-023-05509-4)
Supplement: Supplementary file 1 — Additional file 1. Supplemental Figures for simulation and real data analysis results. [file 12859_2023_5509_MOESM1_ESM.pdf]

Supplemental Material for "Cross-study analyses of microbial abundance using generalized common factor methods"

Molly G. Hayes, Morgan G. I. Langille, and Hong Gu

Additional Simulation Results

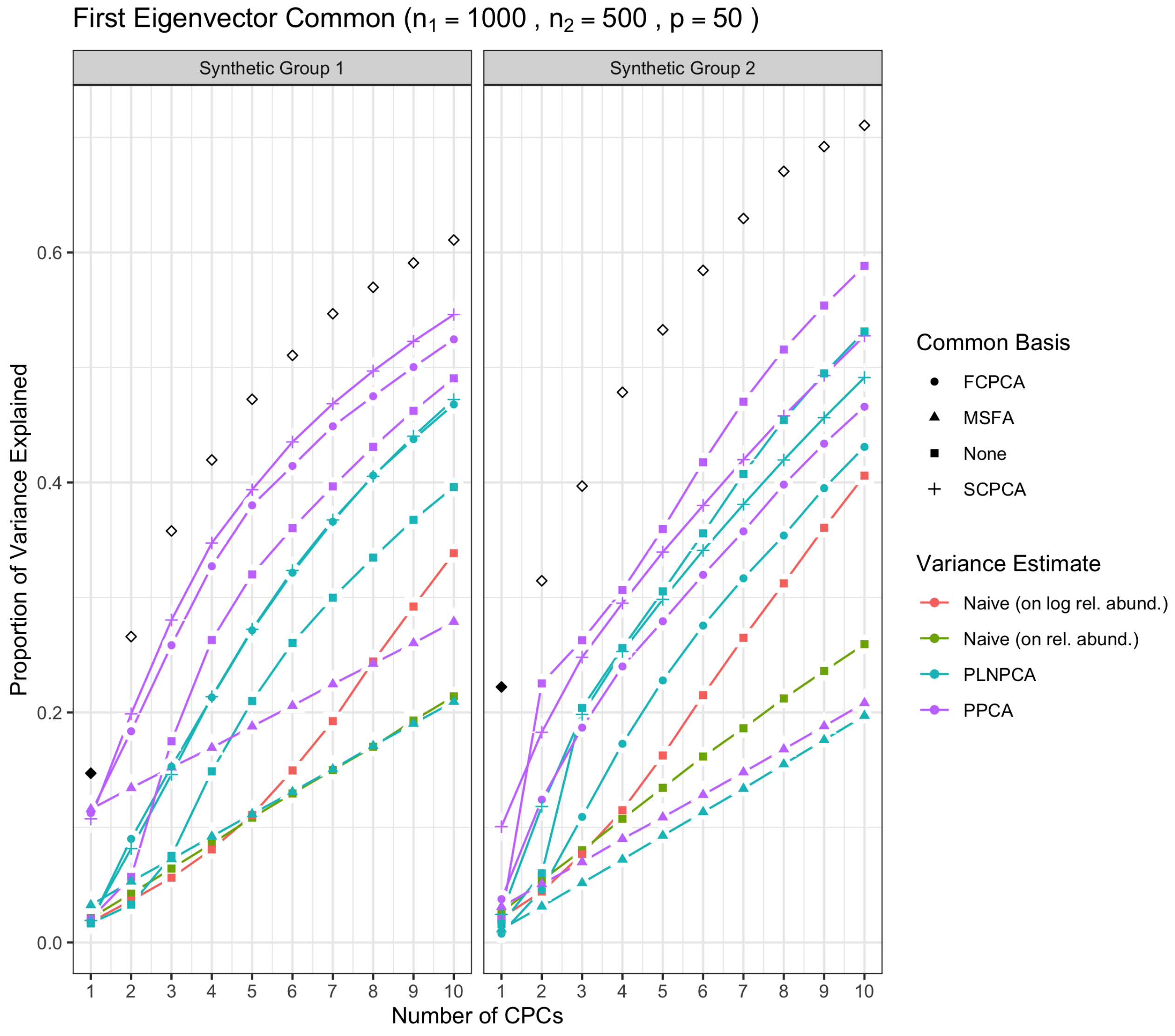

Figure S1: Simulation results for decreasing eigenvalues and one common eigenvector, with sequencing depth correction; p=50, n_1 = 1000, n_2 = 500. "None" as a common basis label means that Group 1 and Group 2 data were concatenated prior to variance estimation. The true common variances are in solid black; the true unique variances are in black outline-only.

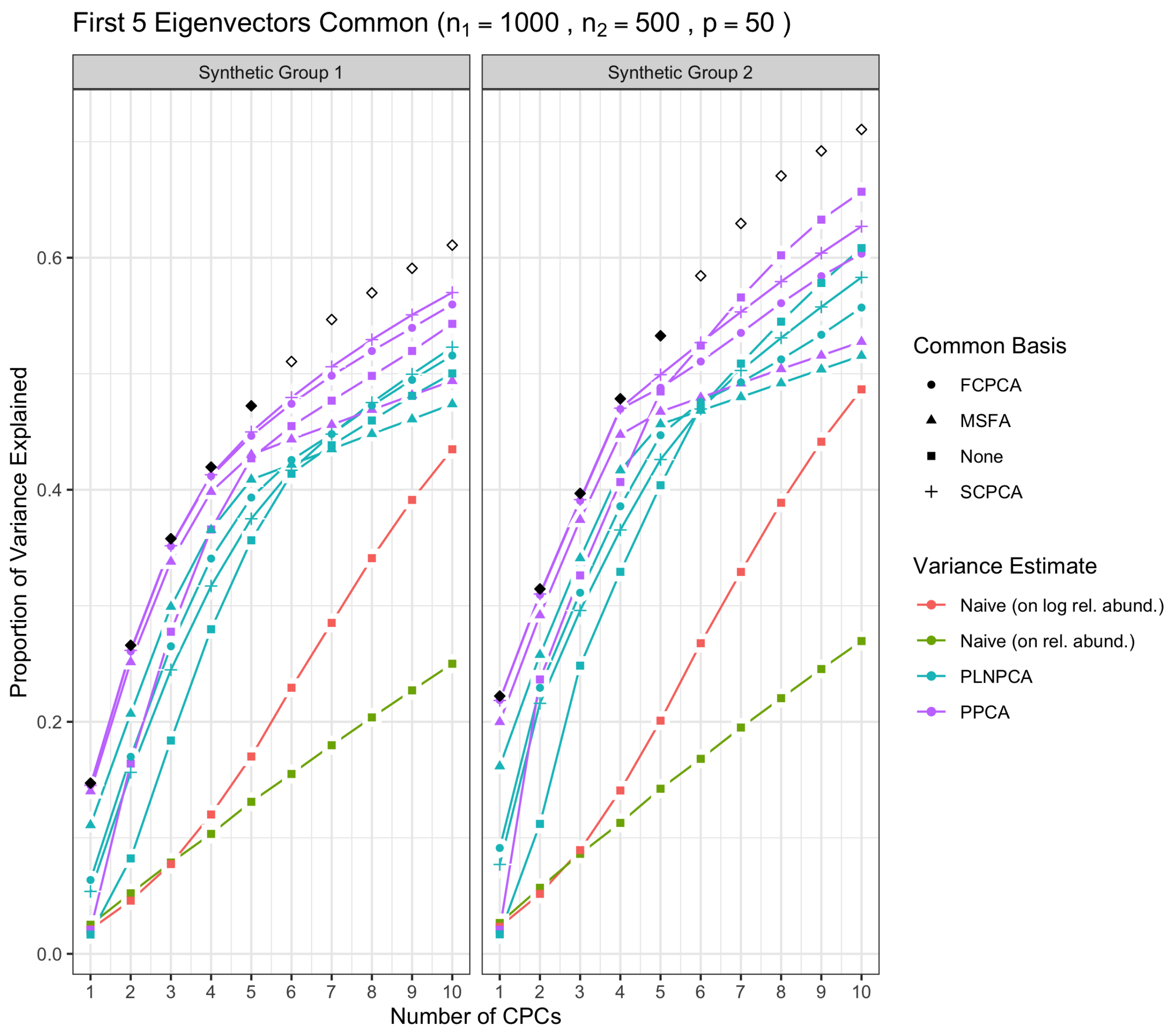

Figure S2: Simulation results for decreasing eigenvalues and five common eigenvectors, with sequencing depth correction; p=50, $n_1 = 1000$, $n_2 = 500$. ”None” as a common basis label means that Group 1 and Group 2 data were concatenated prior to variance estimation. The true common variances are in solid black; the true unique variances are in black outline-only.

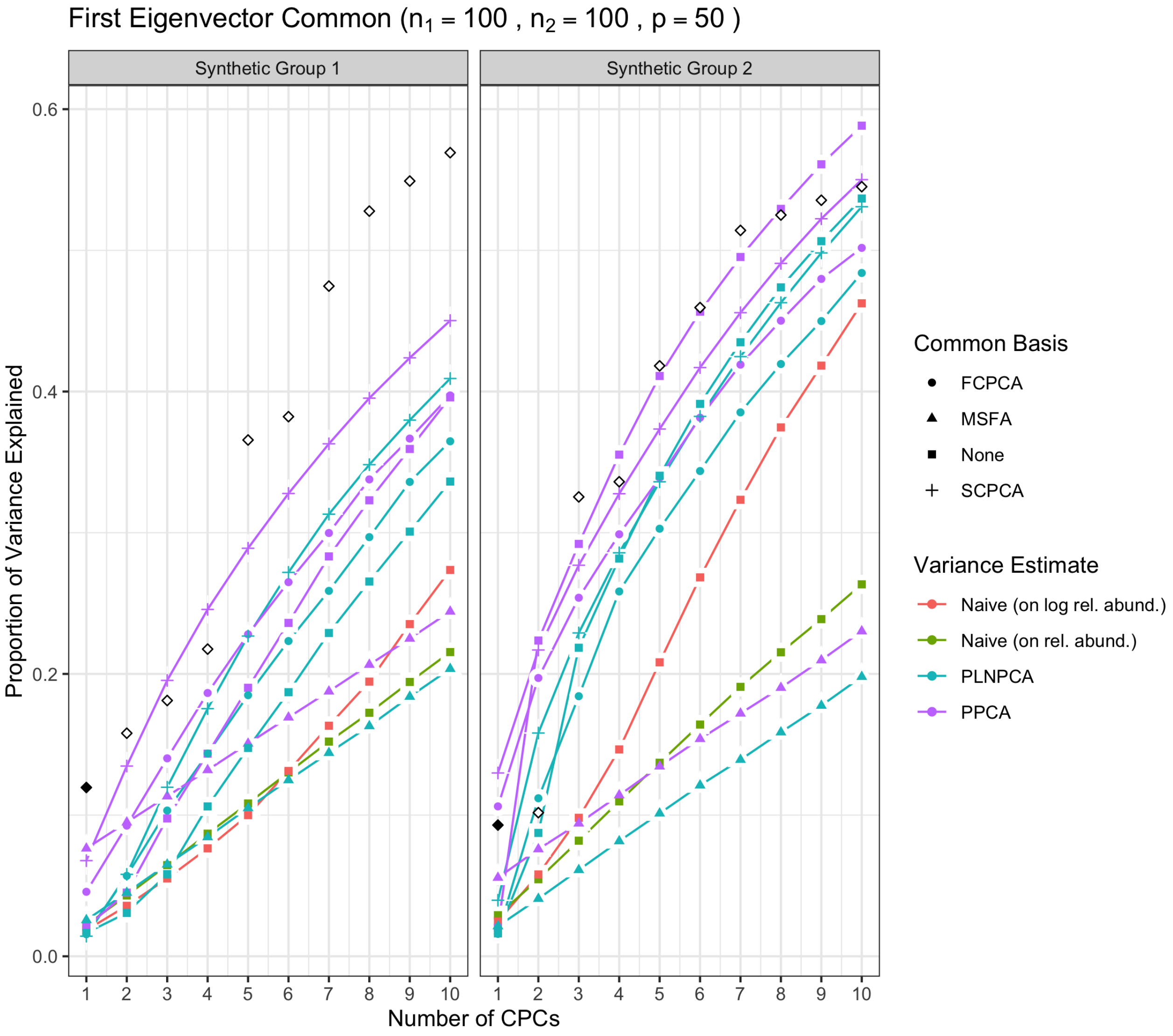

Figure S3: Simulation results for non-decreasing eigenvalues and one common eigenvector, with sequencing depth correction; p=50, $n_1 = 100$, $n_2 = 100$. "None" as a common basis label means that Group 1 and Group 2 data were concatenated prior to variance estimation. The true common variances are in solid black; the true unique variances are in black outline-only.

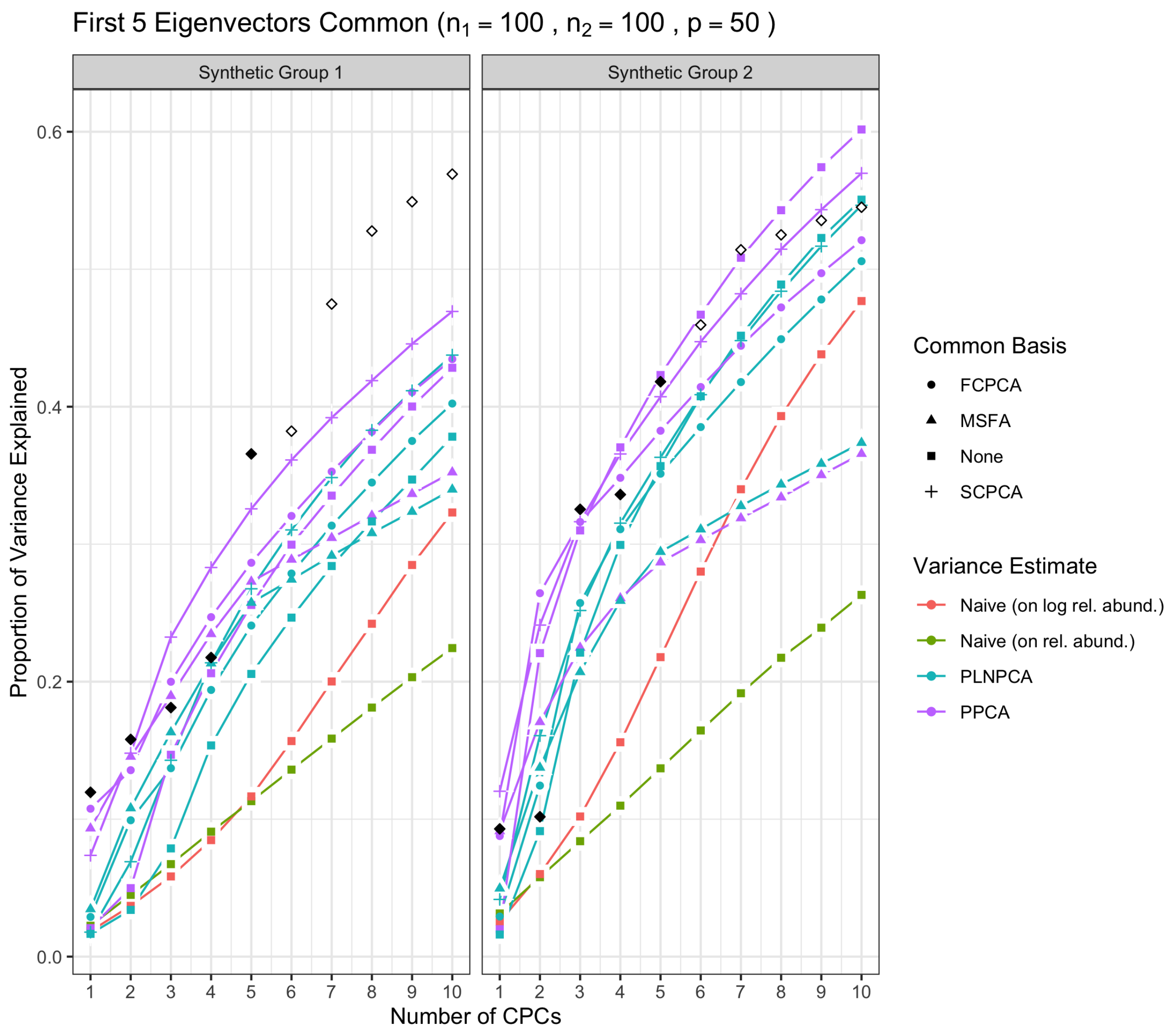

Figure S4: Simulation results for non-decreasing eigenvalues and five common eigenvectors, with sequencing depth correction; p=50, $n_1 = 100$, $n_2 = 100$. "None" as a common basis label means that Group 1 and Group 2 data were concatenated prior to variance estimation. The true common variances are in solid black; the true unique variances are in black outline-only.

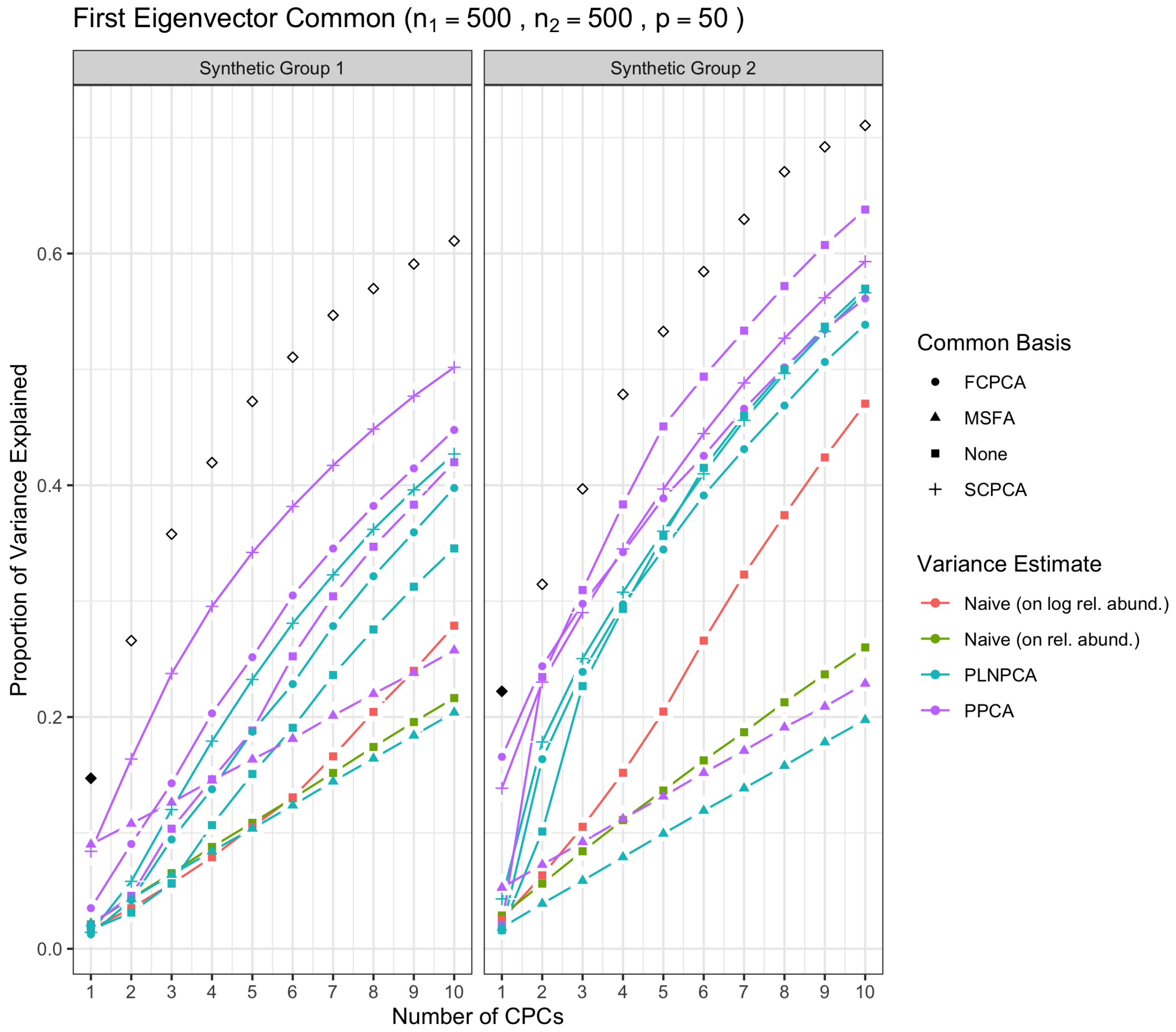

Figure S5: Simulation results for decreasing eigenvalues and one common eigenvector, with sequencing depth correction; p=50, $n_1 = 500$, $n_2 = 500$. "None" as a common basis label means that Group 1 and Group 2 data were concatenated prior to variance estimation. The true common variances are in solid black; the true unique variances are in black outline-only.

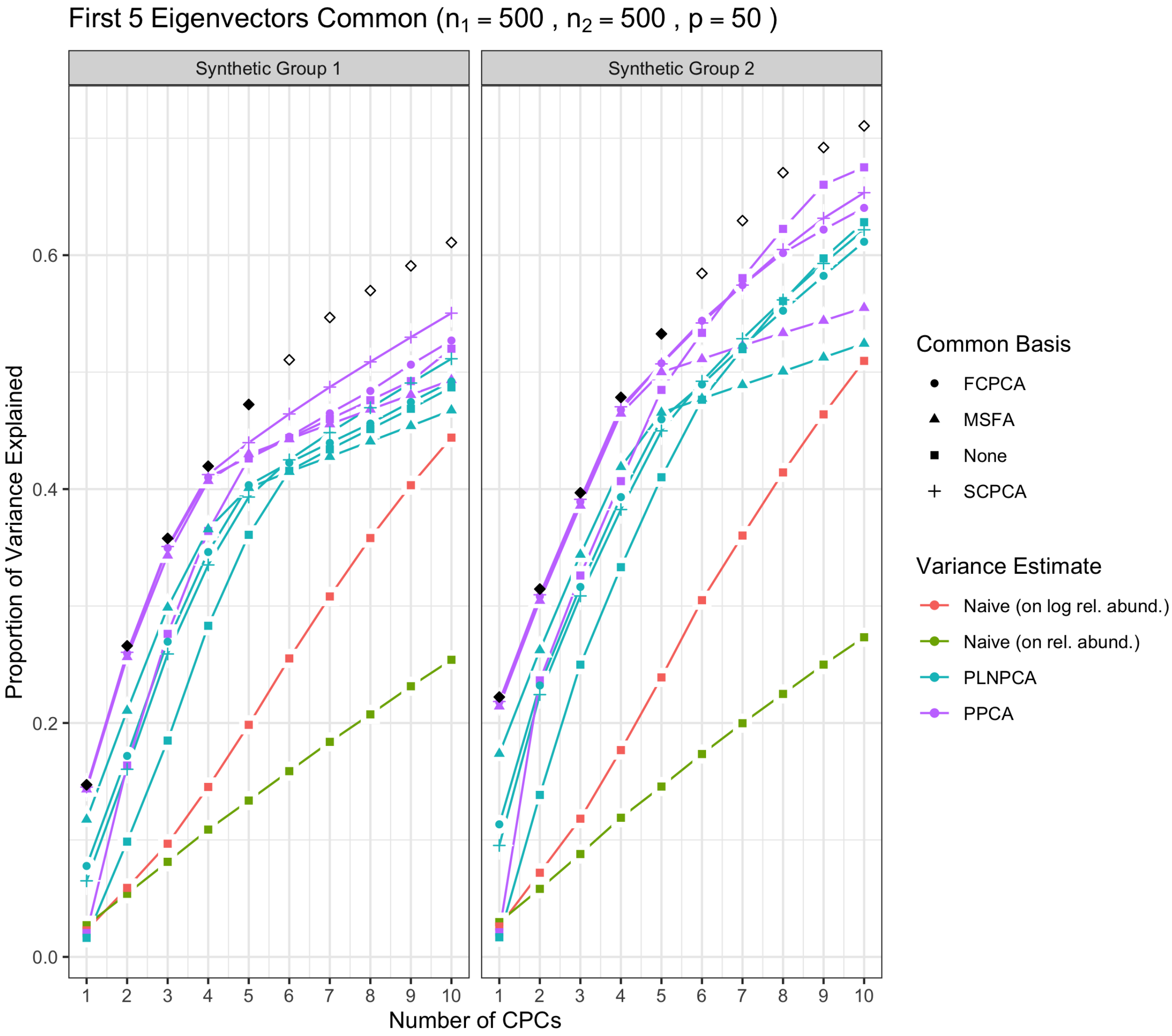

Figure S6: Simulation results for decreasing eigenvalues and five common eigenvectors, with sequencing depth correction; p=50, $n_1 = 200$, $n_2 = 500$. "None" as a common basis label means that Group 1 and Group 2 data were concatenated prior to variance estimation. The true common variances are in solid black; the true unique variances are in black outline-only.

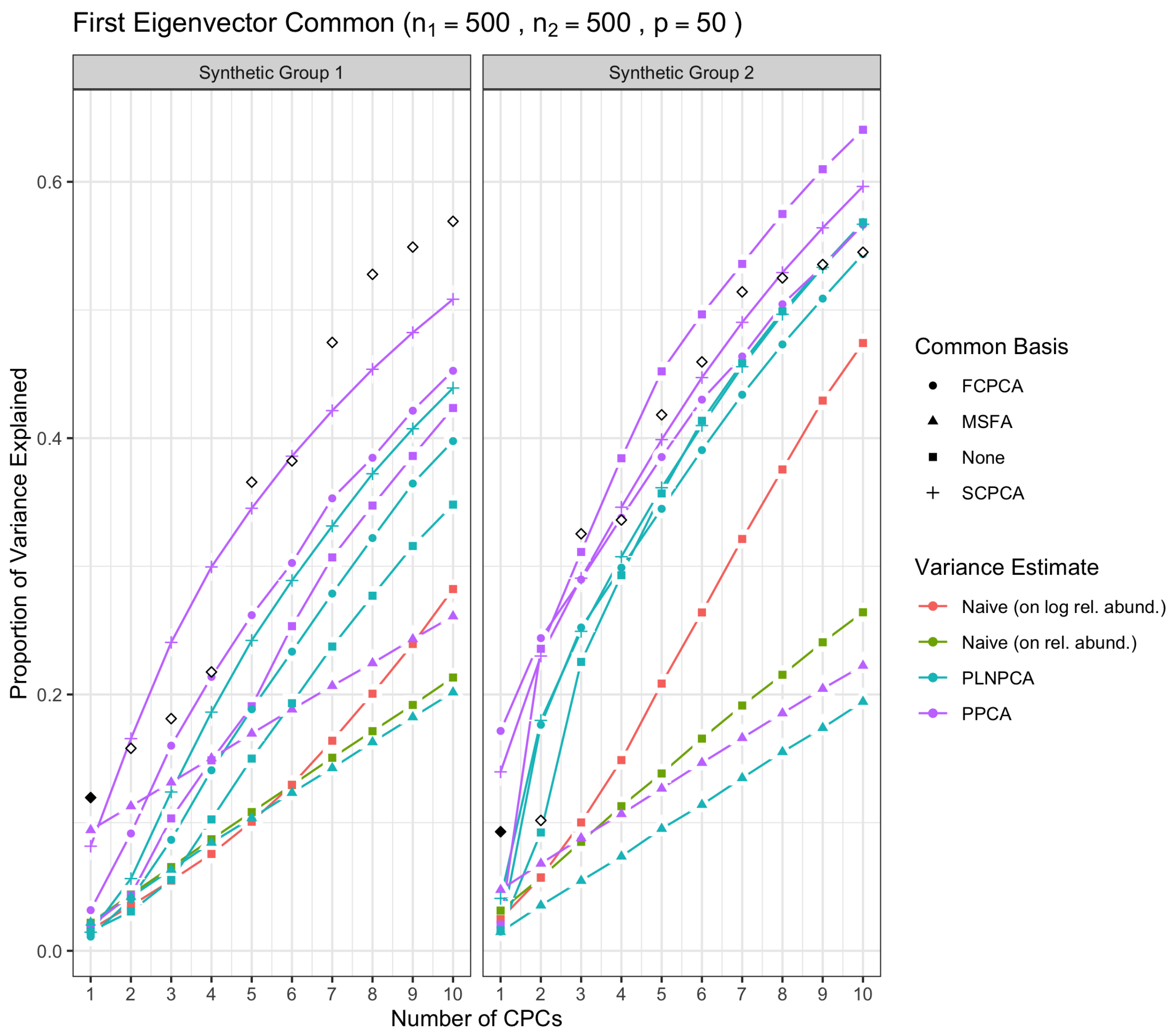

Figure S7: Simulation results for non-decreasing eigenvalues and one common eigenvector, with SDC; p=50, $n_1 = 500$, $n_2 = 500$. ”None” as a common basis label means that Group 1 and Group 2 data were concatenated prior to variance estimation. The true common variances are in solid black; the true unique variances are in black outline-only.

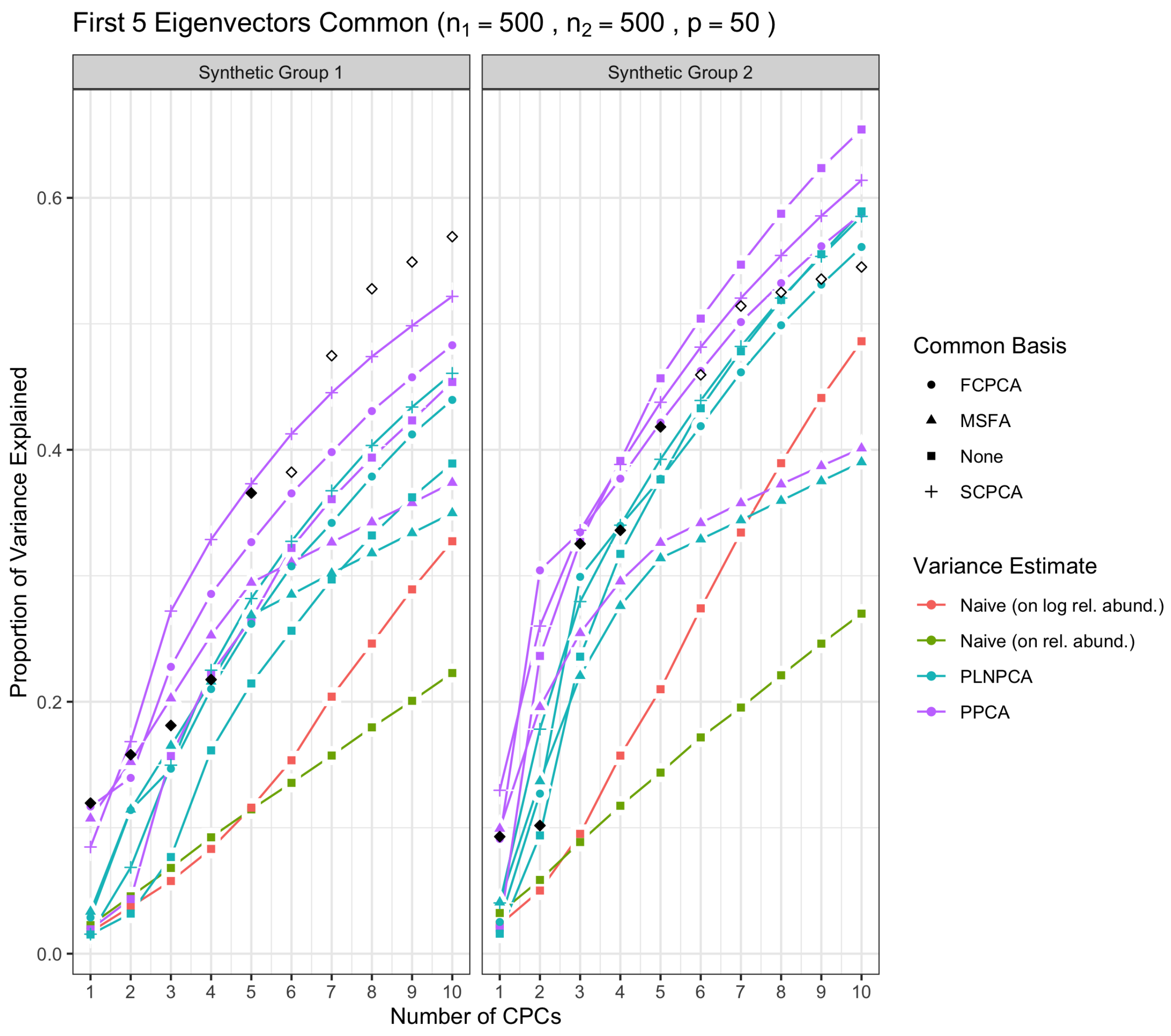

Figure S8: Simulation results for non-decreasing eigenvalues and five common eigenvectors, with SDC; p=50, $n_1 = 500$, $n_2 = 500$. "None" as a common basis label means that Group 1 and Group 2 data were concatenated prior to variance estimation. The true common variances are in solid black; the true unique variances are in black outline-only.

Additional Score Plots

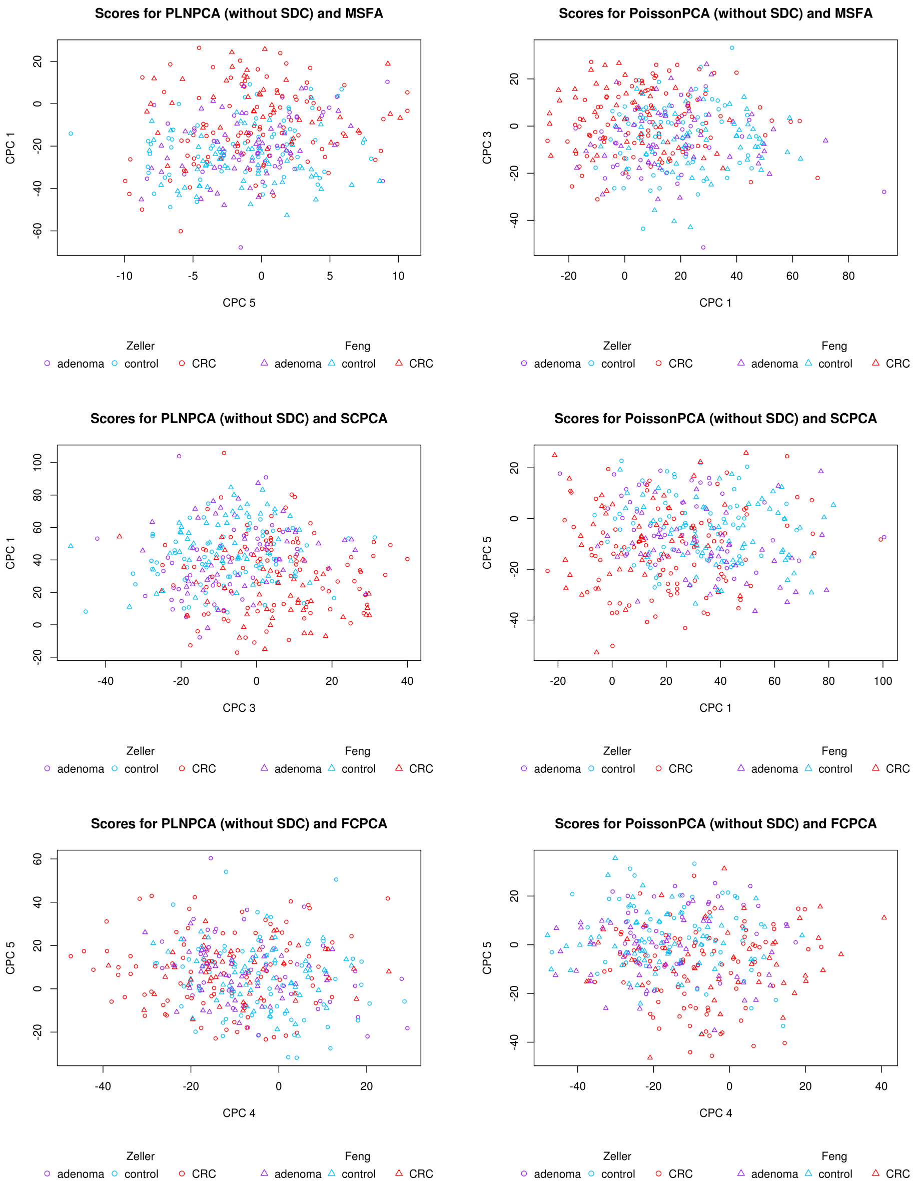

Figure S9: Scores from ensemble methods without SDC by disease state.

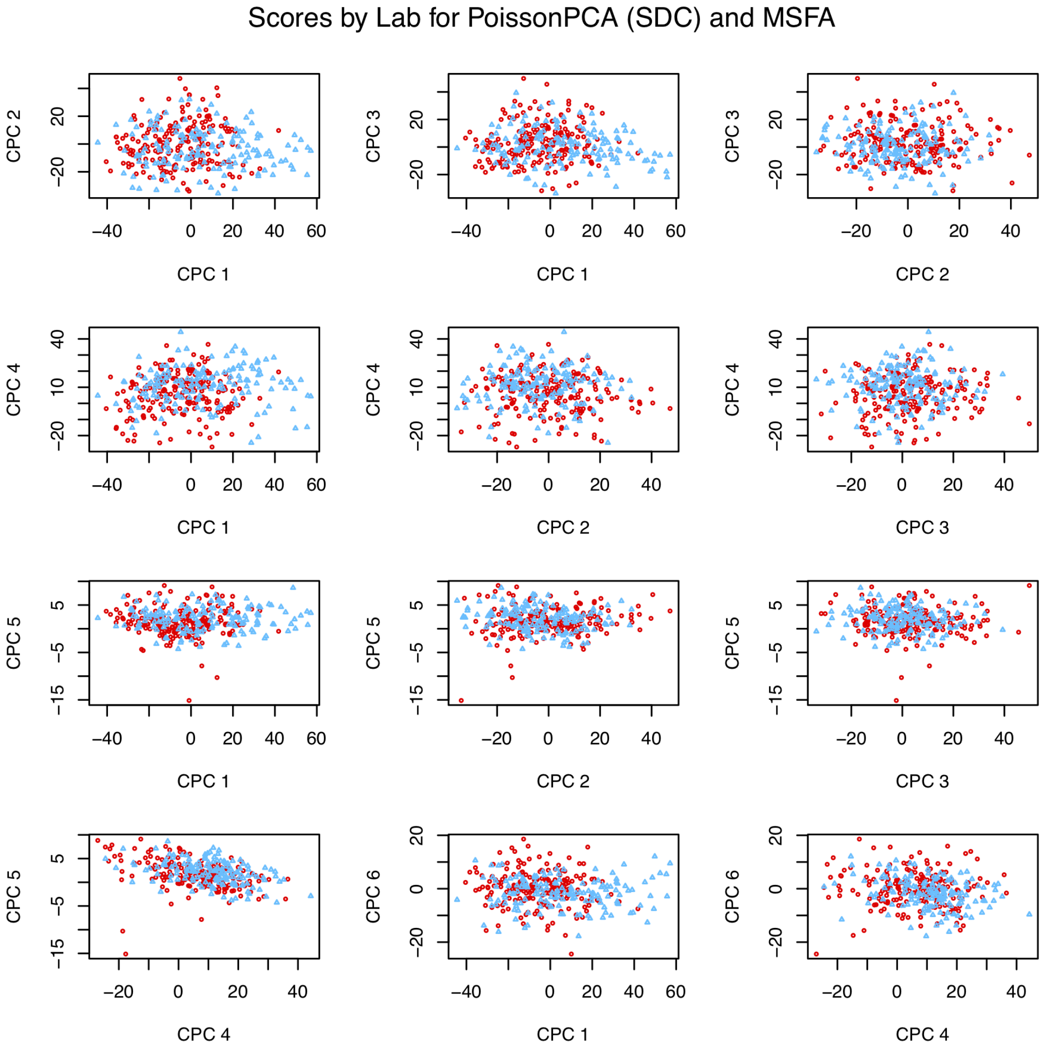

Figure S10: Scores from PoissonPCA (SDC) and MSFA by study of origin.

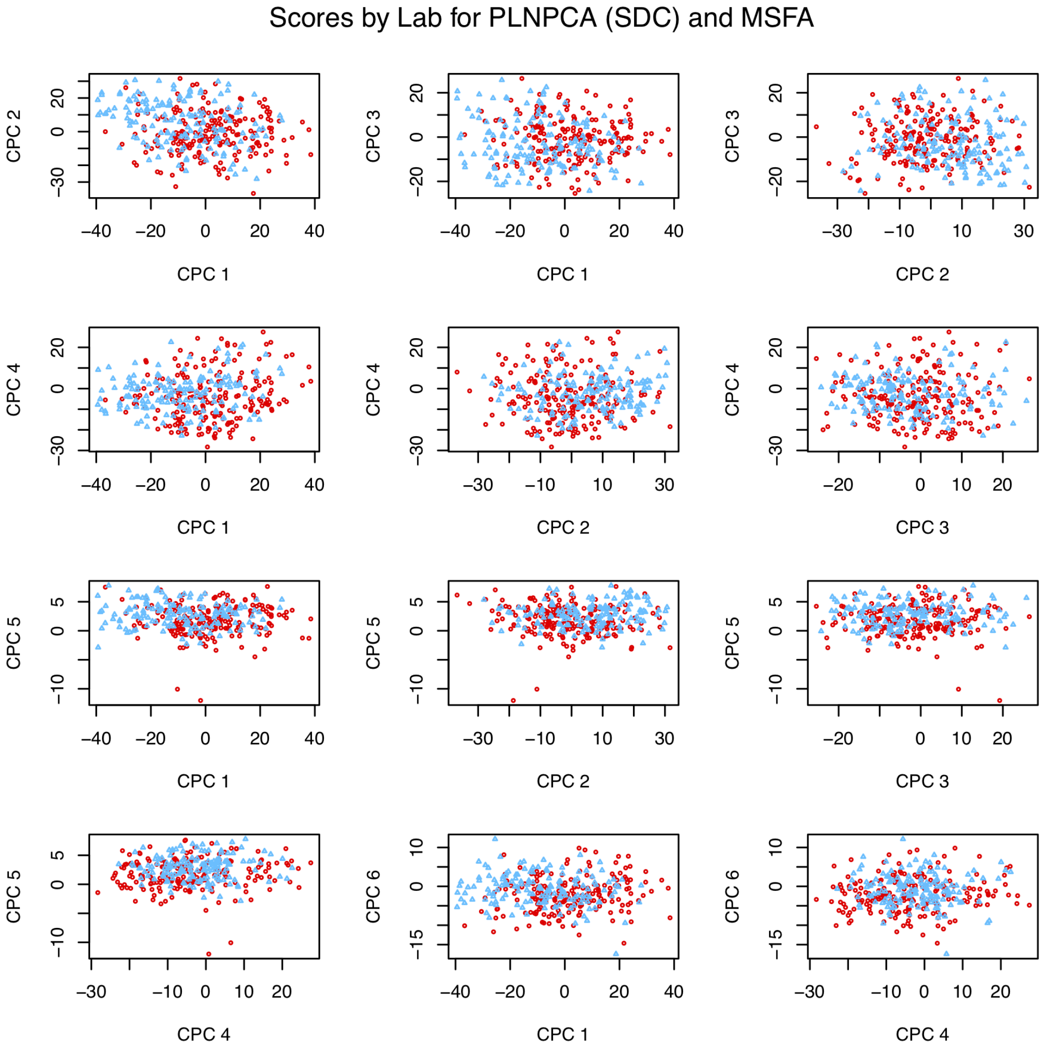

Figure S11: Scores from PLNPCA (SDC) and MSFA by study of origin.

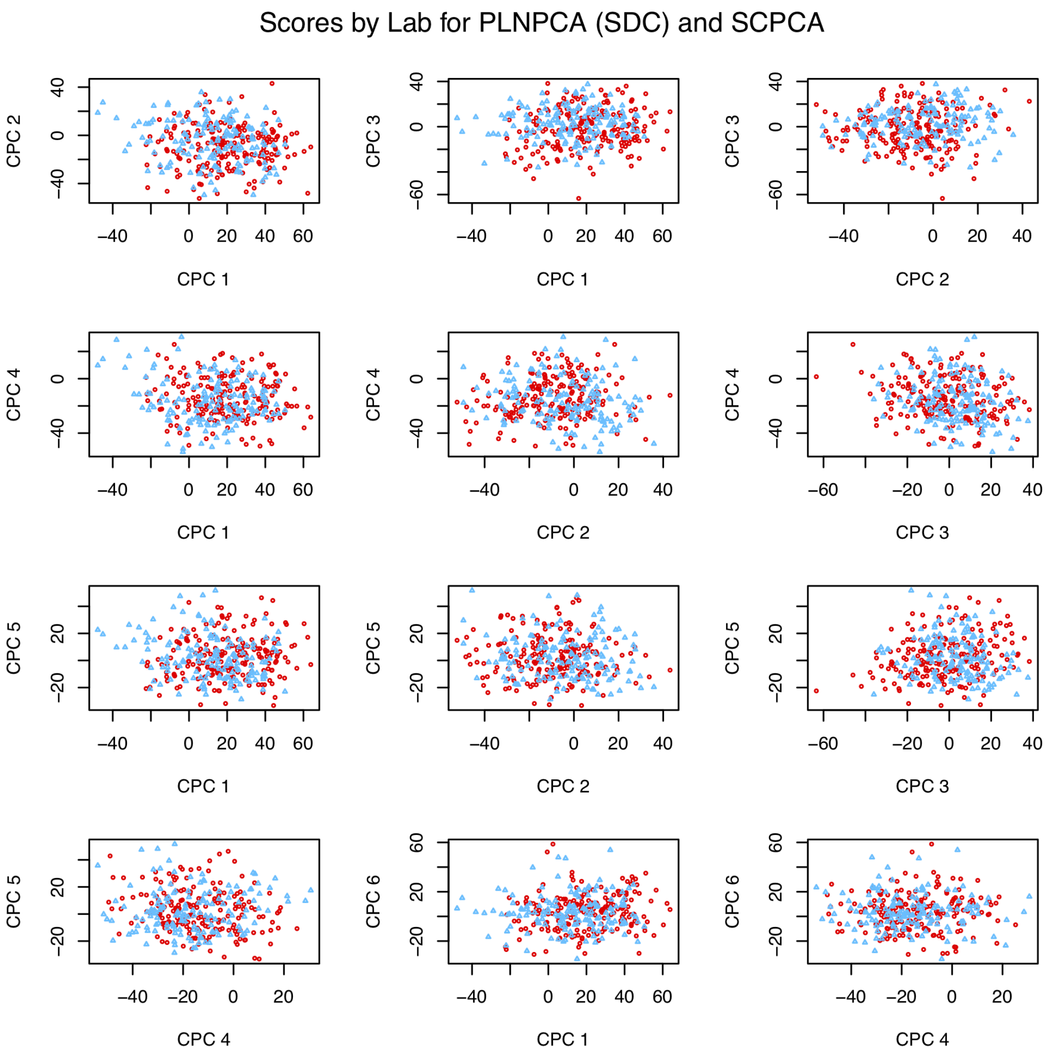

Figure S12: Scores from PLNPCA (SDC) and SCPCA by study of origin.

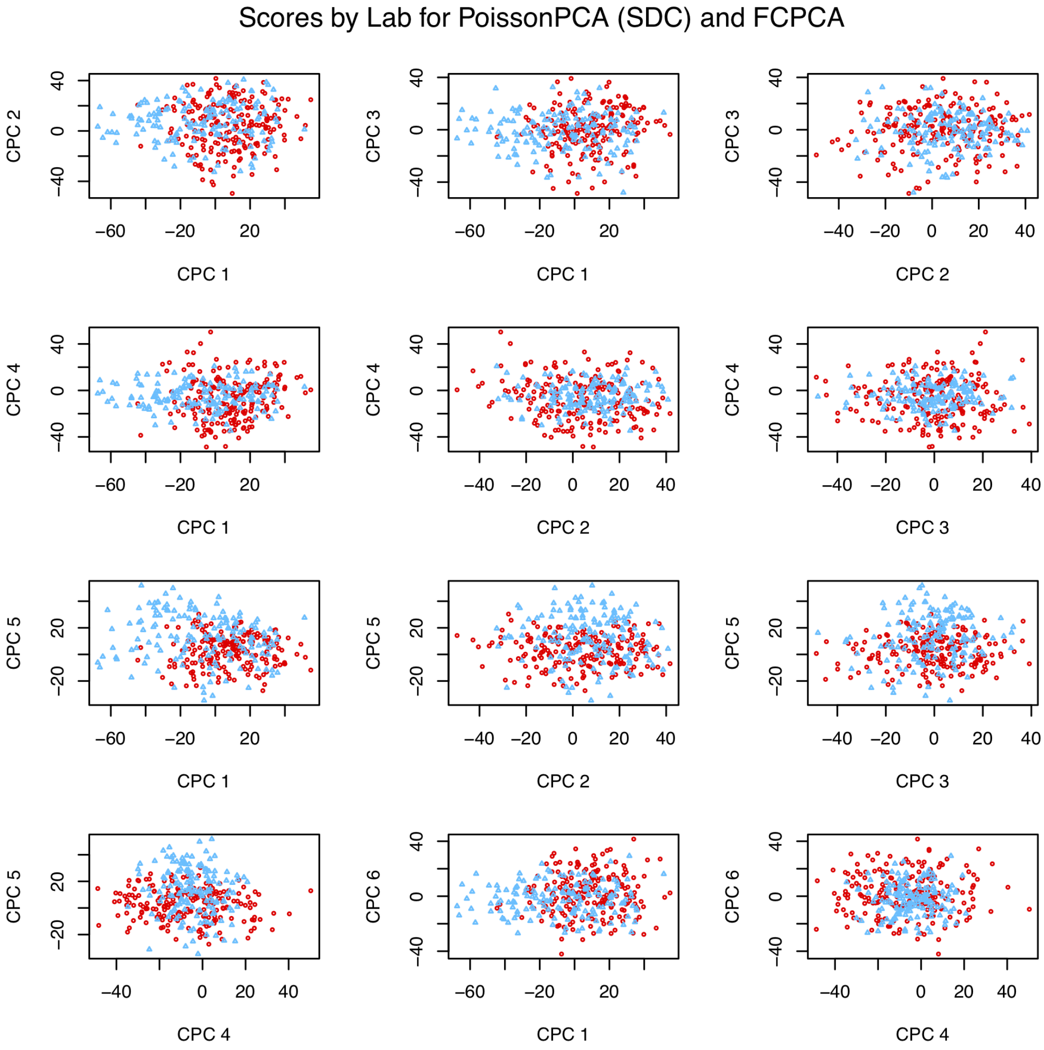

Figure S13: Scores from PoissonPCA (SDC) & FCPCA by study of origin.

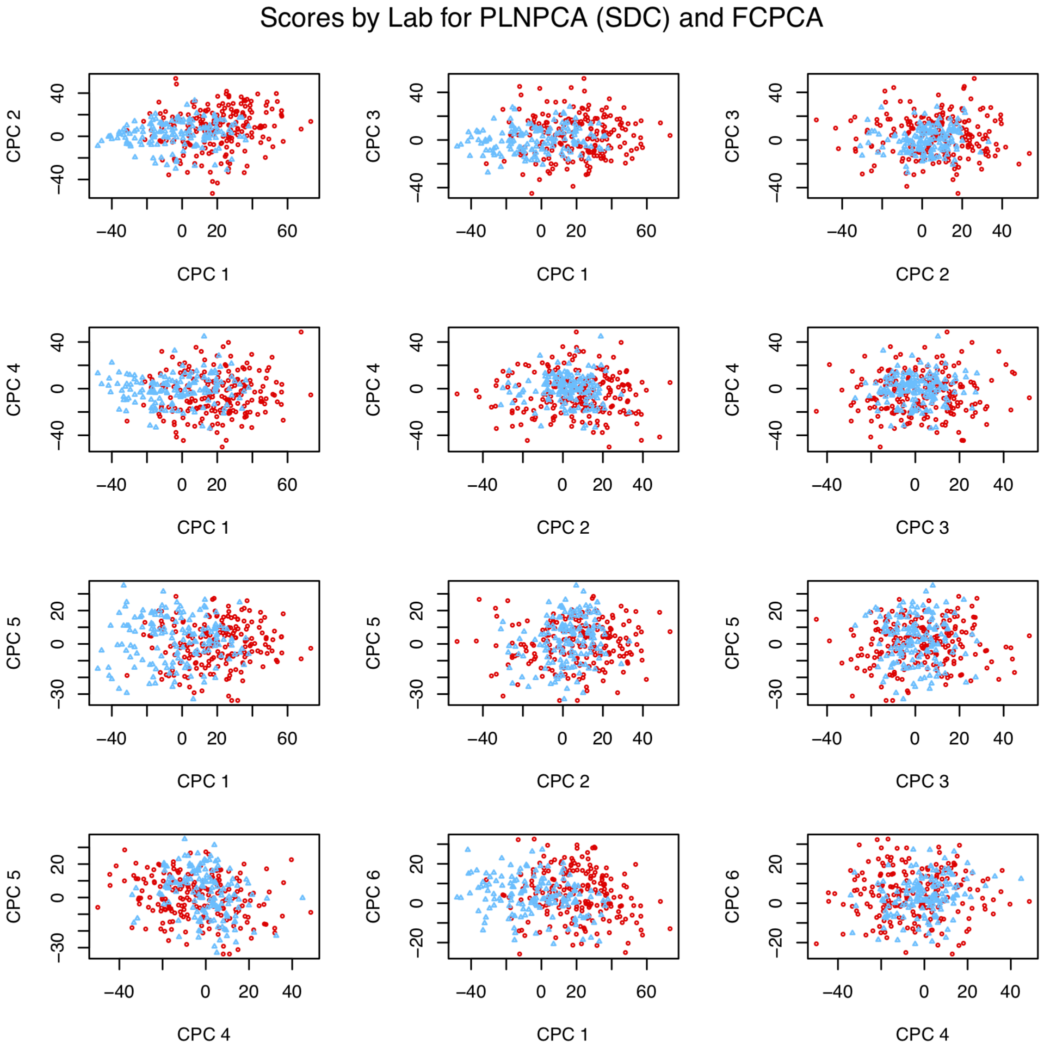

Figure S14: Scores from PLNPCA (SDC) and FCPCA by study of origin.

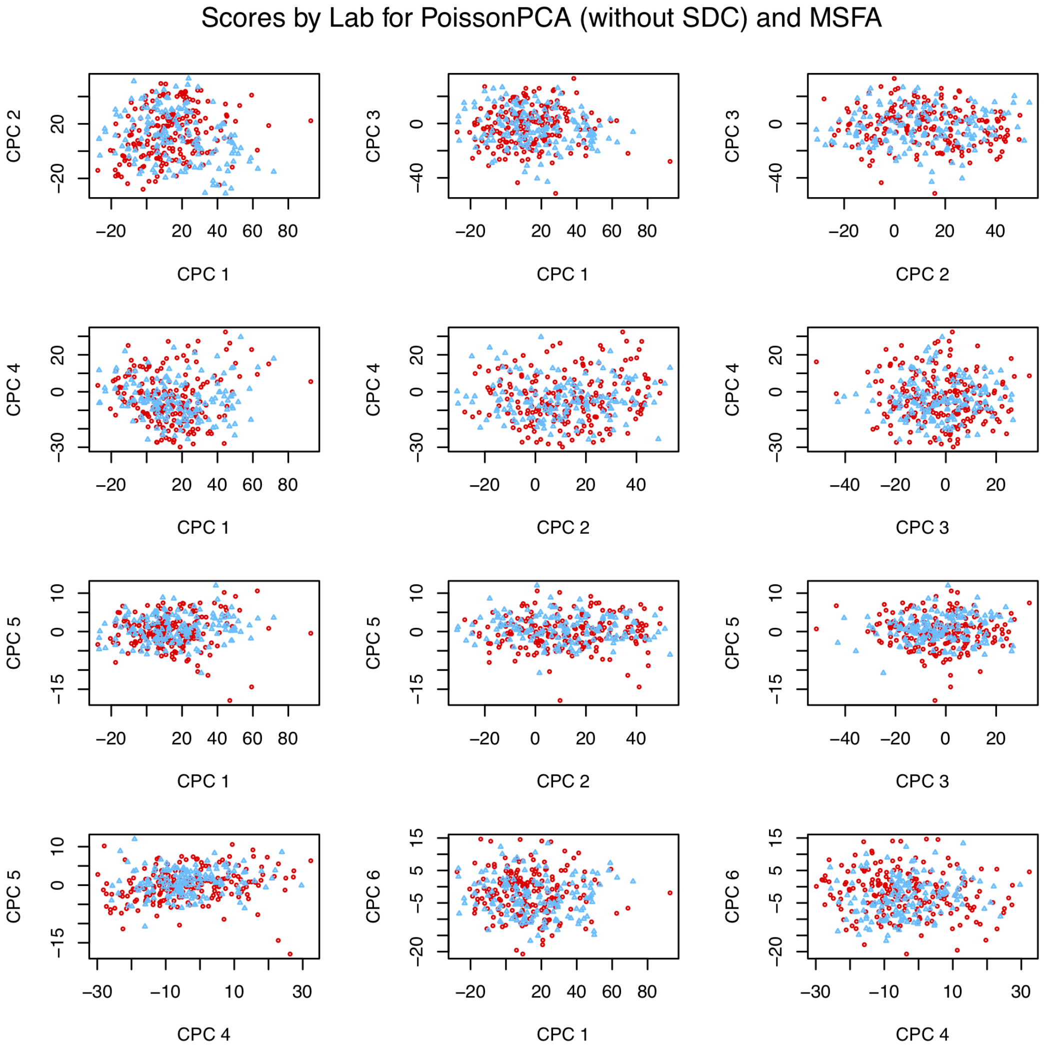

Figure S15: Scores from PoissonPCA (no SDC) & MSFA by study of origin.

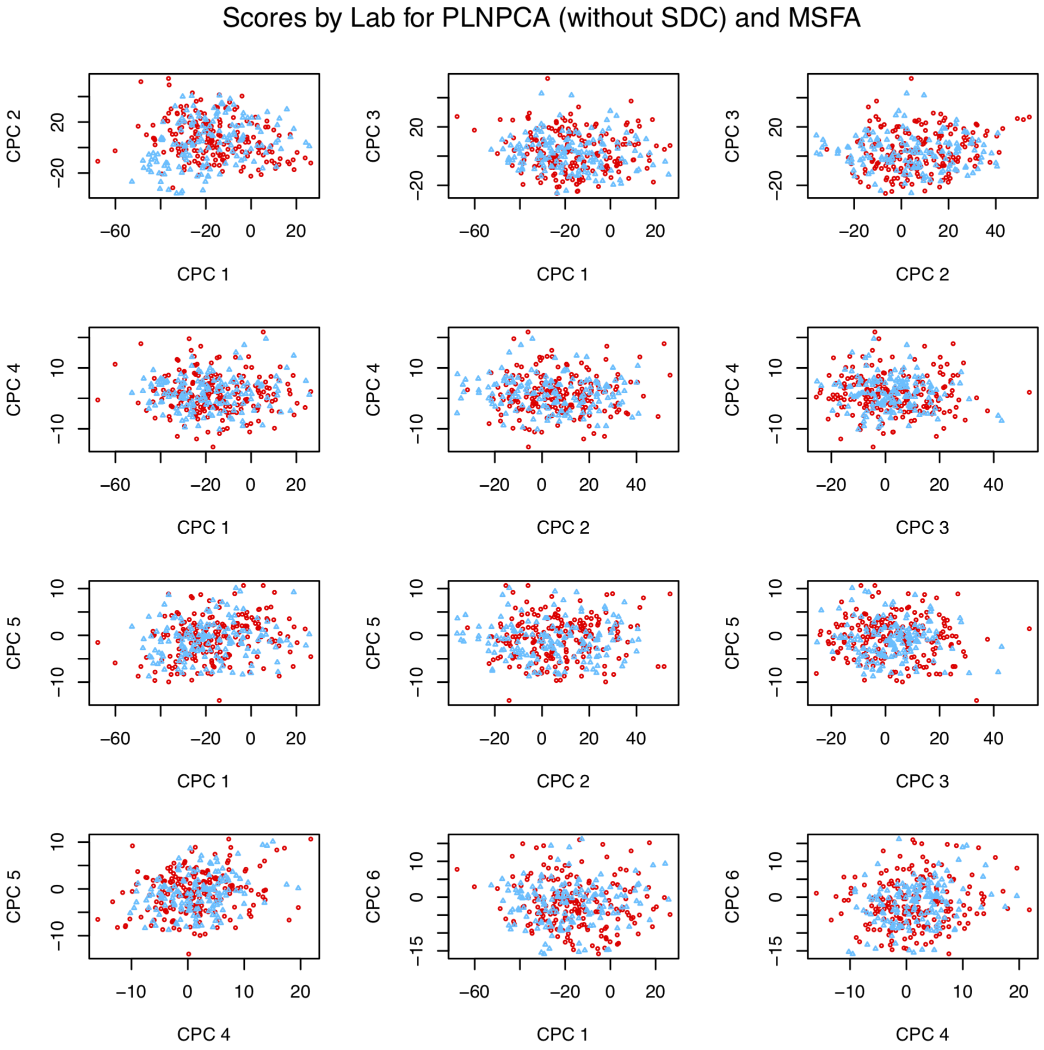

Figure S16: Scores from PLNPCA (no SDC) and MSFA by study of origin.

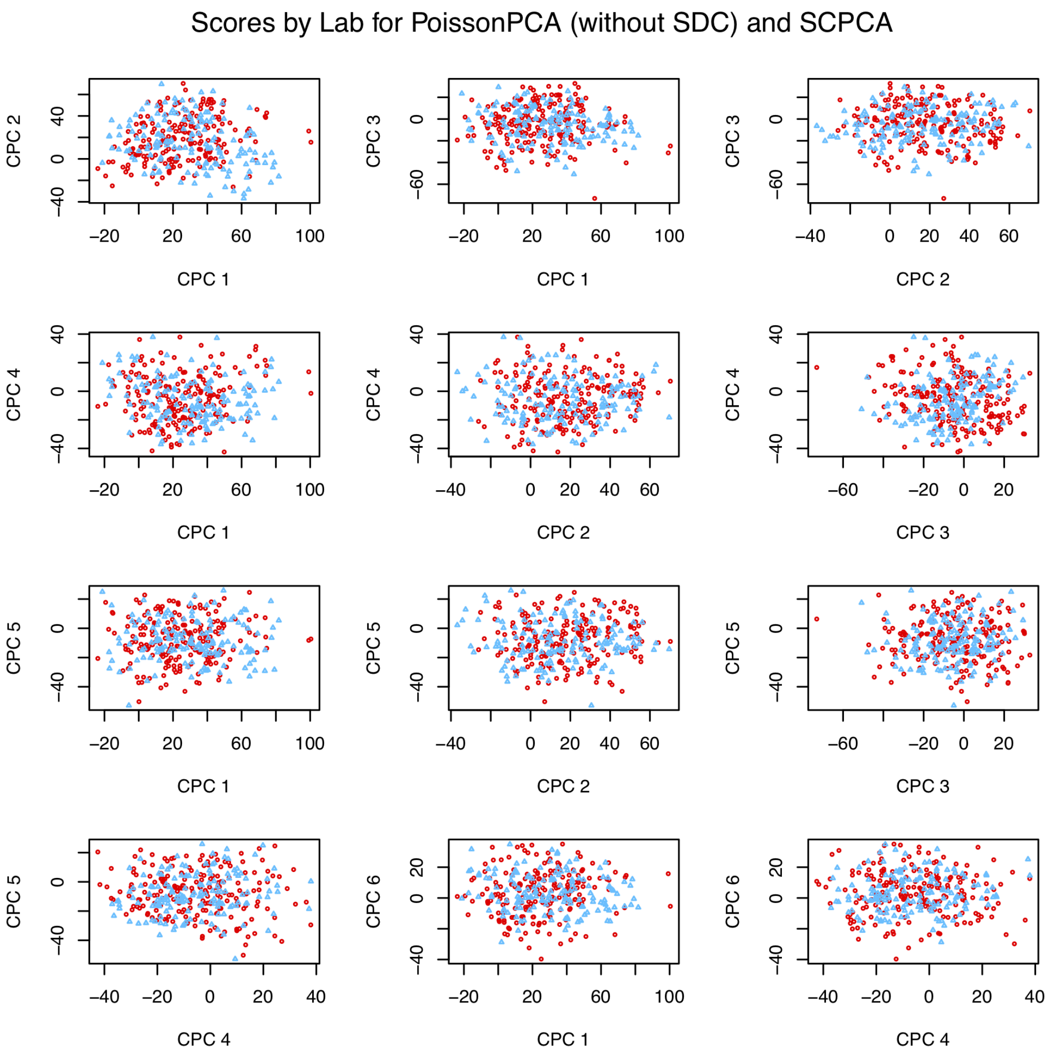

Figure S17: Scores from PoissonPCA (no SDC) & SCPCA by study of origin.

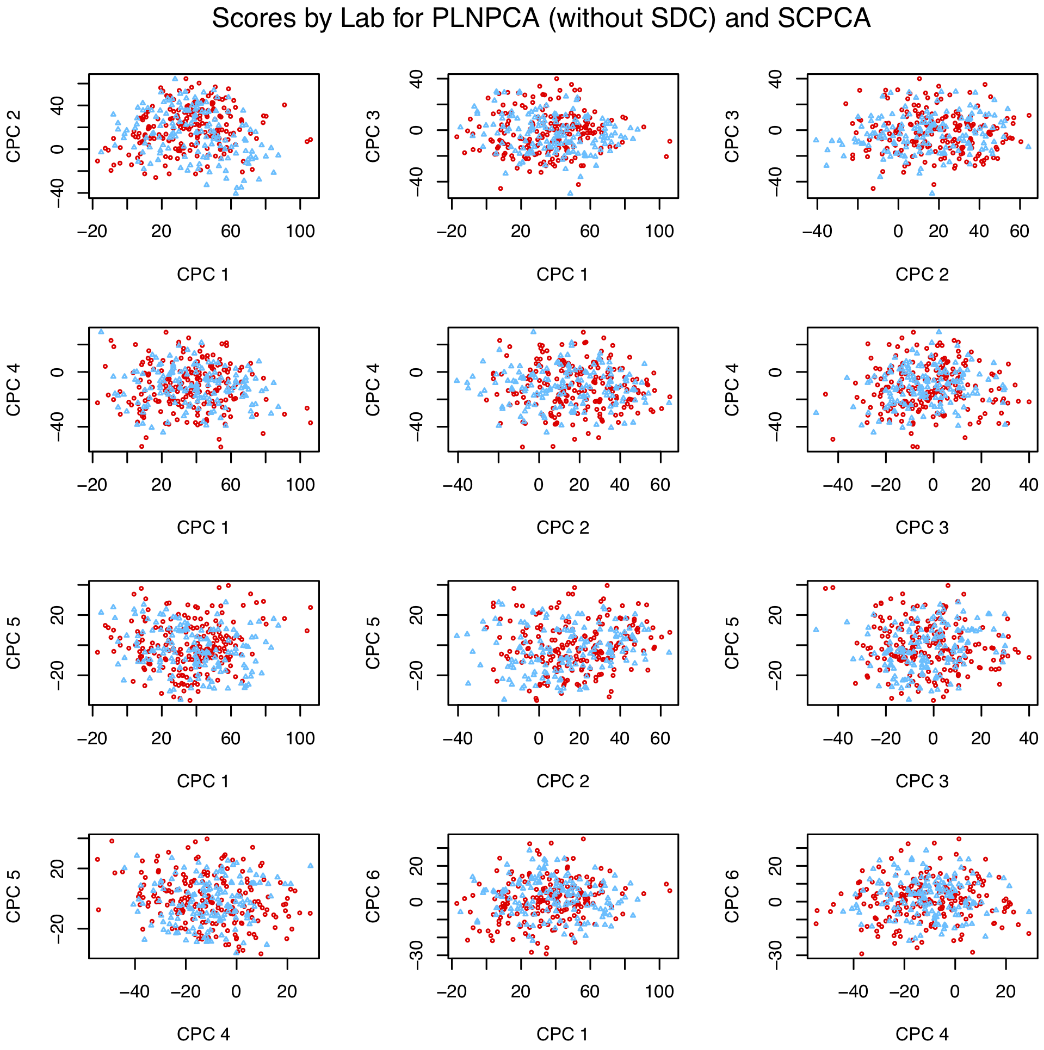

Figure S18: Scores from PLNPCA (no SDC) & SCPCA by study of origin.

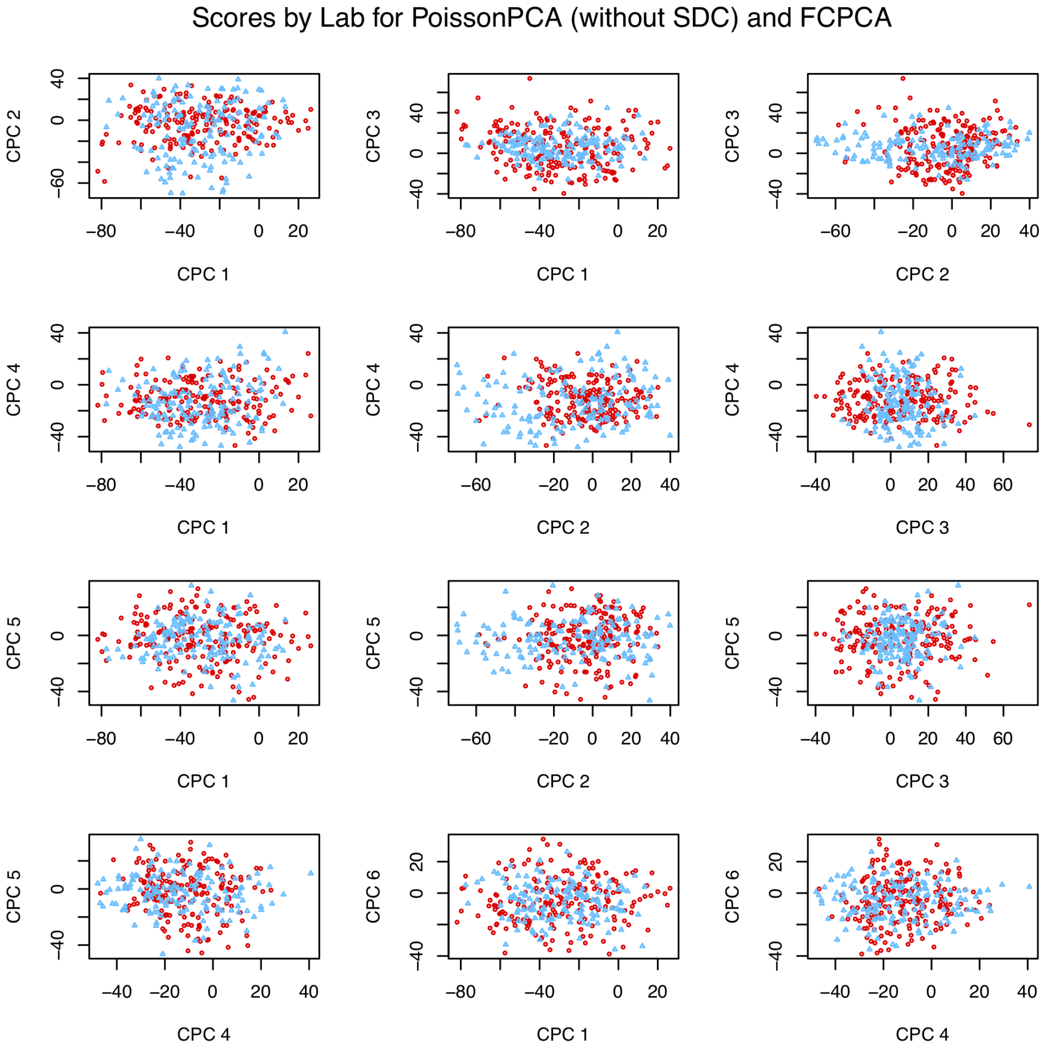

Figure S19: Scores from PoissonPCA (no SDC) & FCPCA by study of origin.

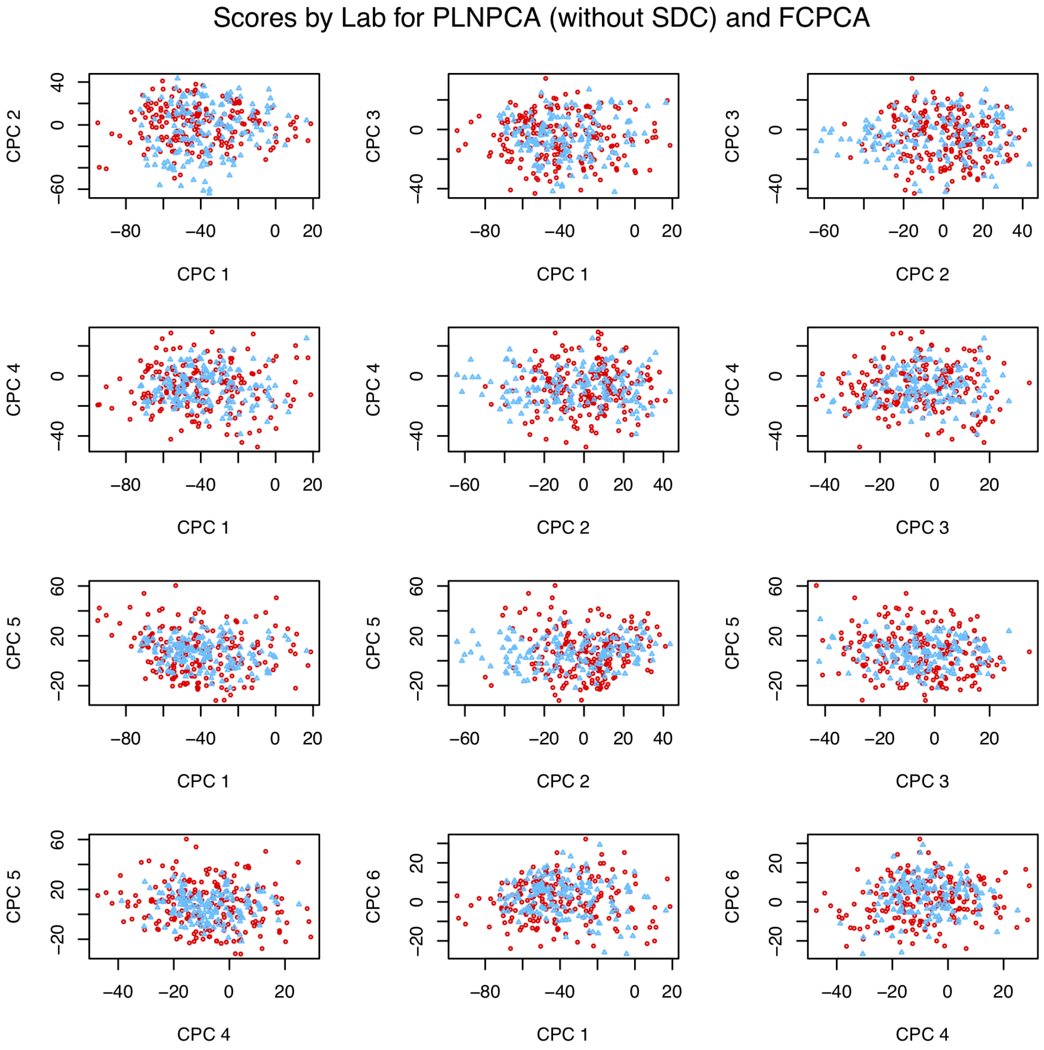

Figure S20: Scores from PLNPCA (no SDC) & FCPCA by study of origin.

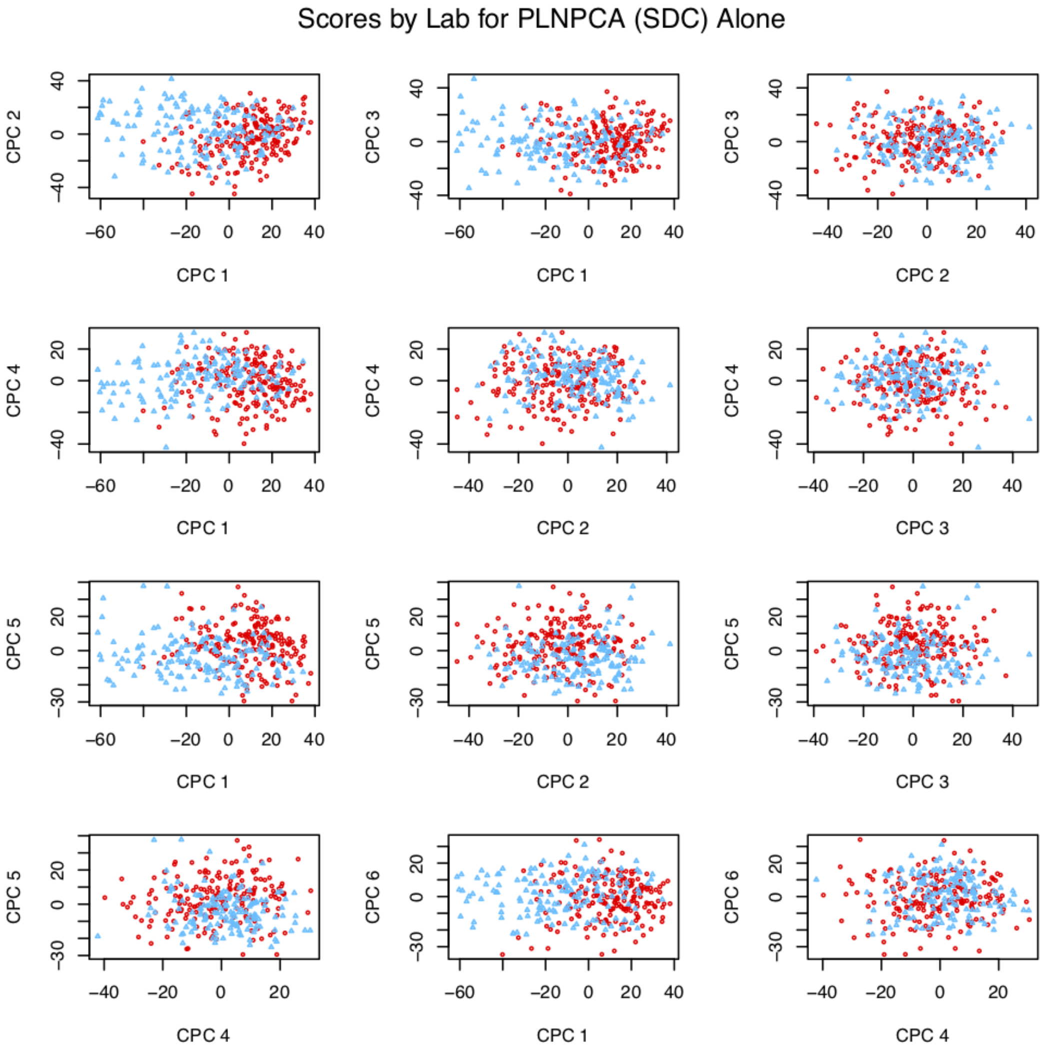

Figure S21: Scores from PLNPCA alone with SDC by study of origin.

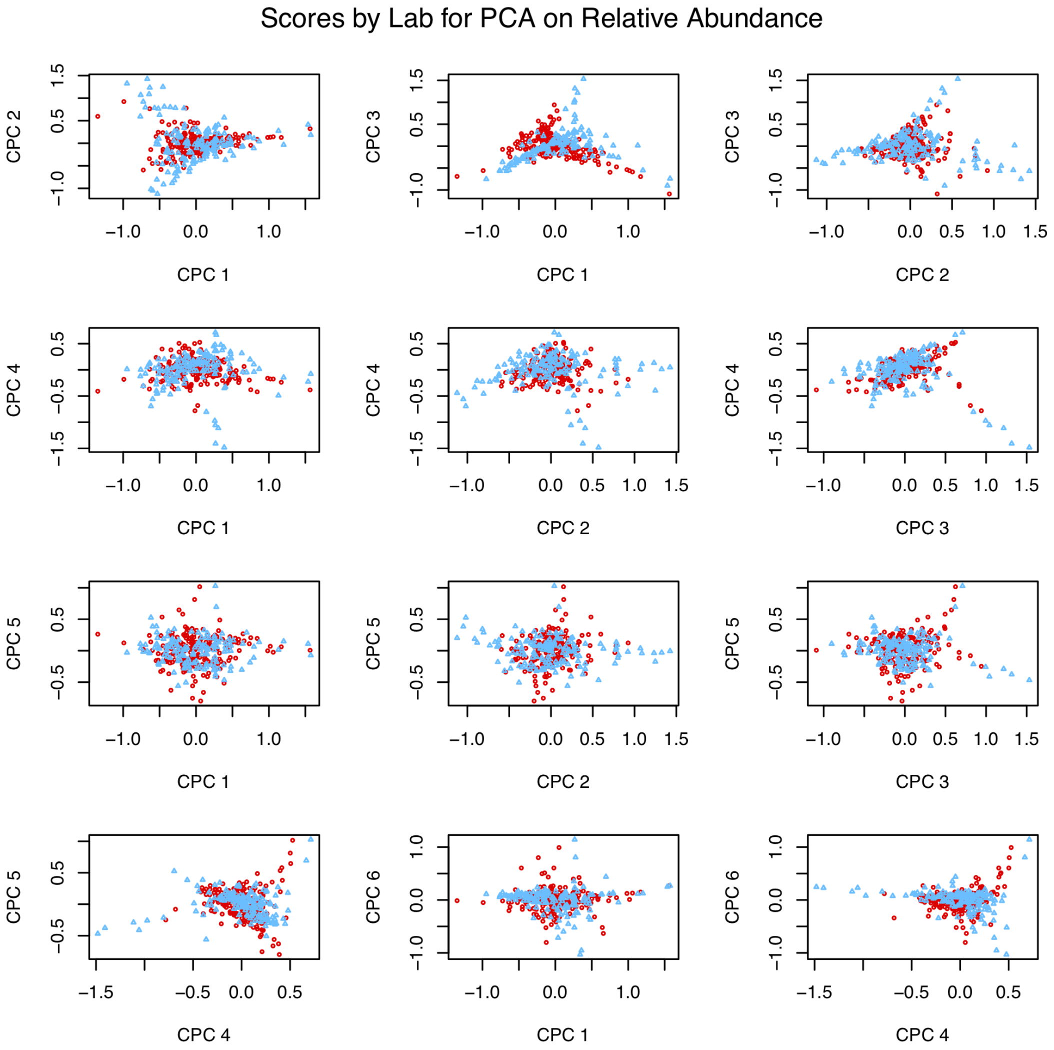

Figure S22: Scores from PCA of relative abundance by study of origin.

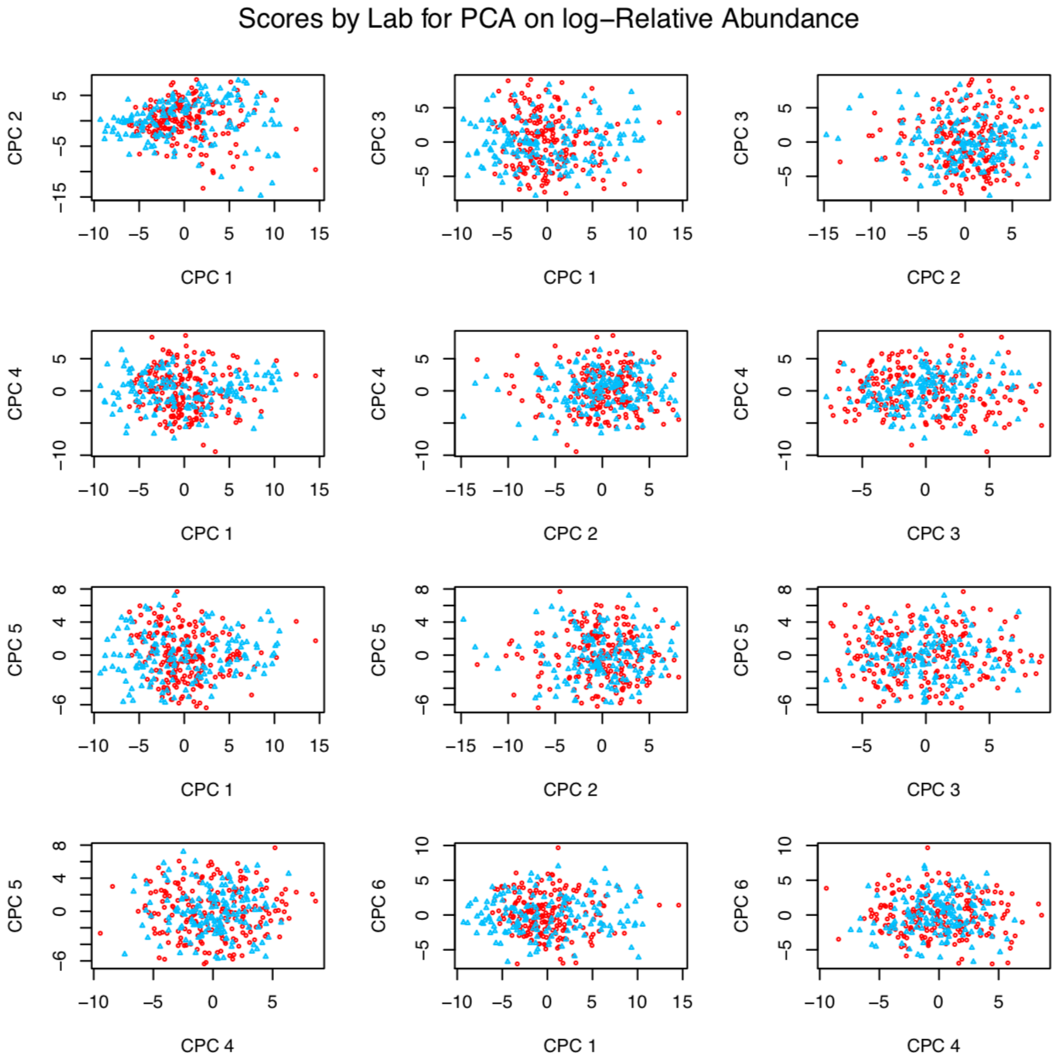

Figure S23: Scores from PCA of log relative abundance by study of origin.

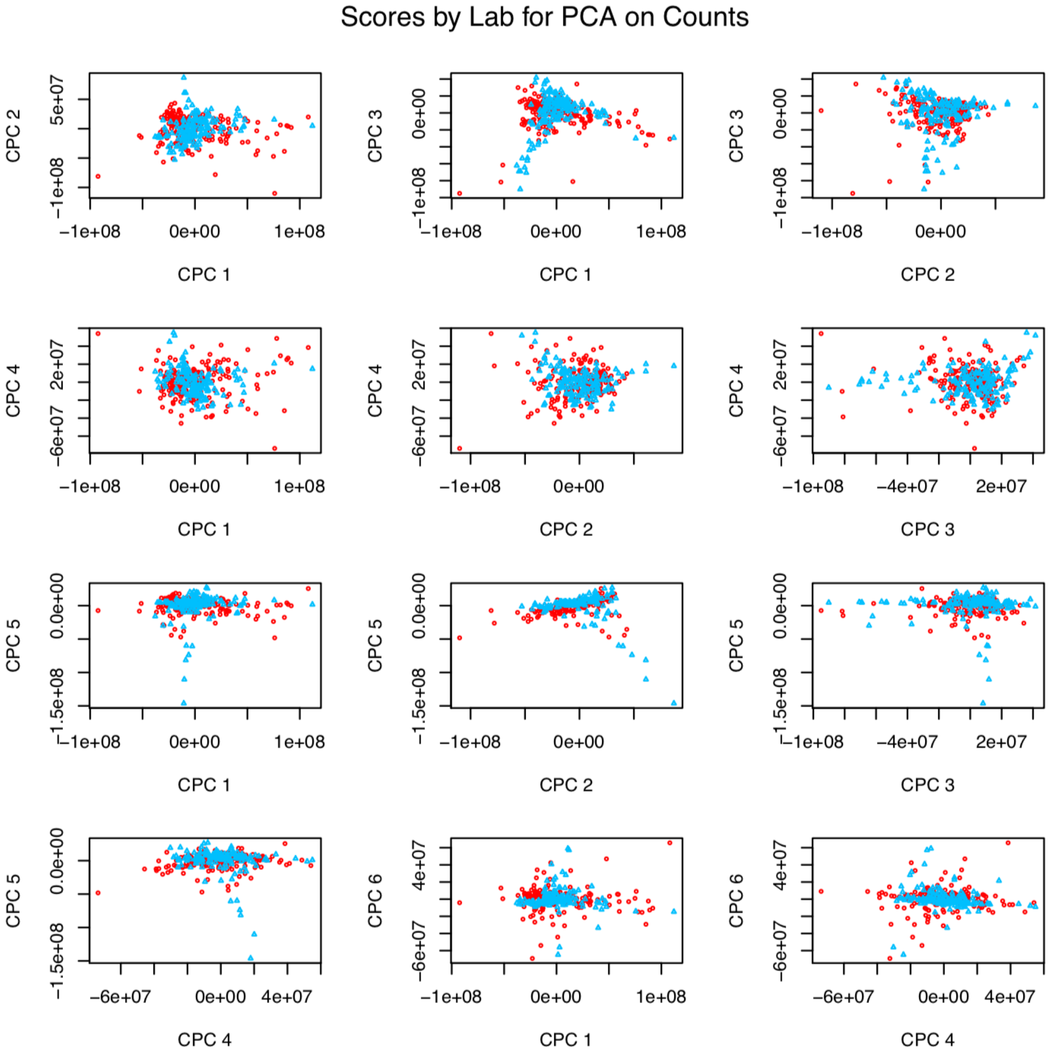

Figure S24: Scores from PCA of counts by study of origin.

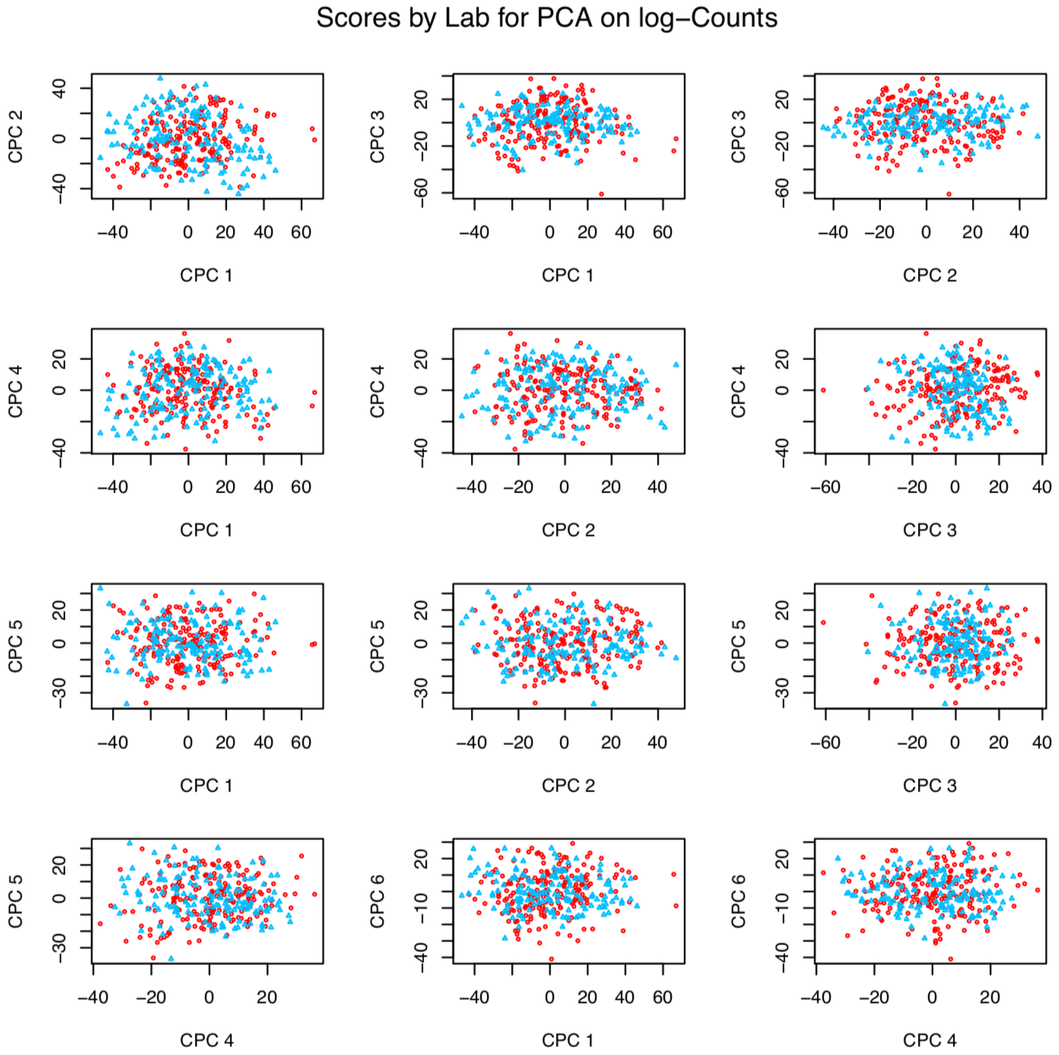

Figure S25: Scores from PCA of log counts by study of origin.
